# Postoperative circulating tumour DNA is associated with pathologic response and recurrence-free survival after resection of colorectal cancer liver metastases

**DOI:** 10.1016/j.ebiom.2021.103498

**Published:** 2021-07-29

**Authors:** Karen Bolhuis, Iris van 't Erve, Clinton Mijnals, Pien M. Delis – Van Diemen, Joost Huiskens, Aysun Komurcu, Marta Lopez-Yurda, Daan van den Broek, Rutger-Jan Swijnenburg, Gerrit A. Meijer, Cornelis J.A. Punt, Remond J.A. Fijneman

**Affiliations:** a Department of Medical Oncology, Amsterdam UMC, Cancer Center Amsterdam, University of Amsterdam, the Netherlands; b Department of Pathology, The Netherlands Cancer Institute, Plesmanlaan 121, Amsterdam 1066CX, the Netherlands; c Department of Pathology, Amphia hospital, Breda, the Netherlands; d SAS Institute B.V., Huizen, the Netherlands; e Netherlands Comprehensive Cancer Center, Utrecht, the Netherlands; f Biometrics Department, The Netherlands Cancer Institute, Amsterdam, the Netherlands; g Department of Clinical Chemistry, The Netherlands Cancer Institute, Amsterdam, the Netherlands; h Department of Surgery, Amsterdam UMC, Cancer Center Amsterdam, University of Amsterdam, the Netherlands; i Department of Epidemiology, Julius Center for Health Sciences and Primary Care, University Medical Center, Utrecht University, Utrecht, the Netherlands

**Keywords:** Colorectal cancer, Liver metastases, Circulating tumour DNA, Resection, Recurrences

## Abstract

**Background:**

Recurrence rates after resection of colorectal cancer liver metastases (CRLM) are high and correlate with worse survival. Postoperative circulating tumour DNA (ctDNA) is a promising prognostic biomarker. Focusing on patients with resected CRLM, this study aimed to evaluate the association between the detection of postoperative ctDNA, pathologic response and recurrence-free survival (RFS).

**Methods:**

Twenty-three patients were selected from an ongoing phase-3 trial who underwent resection of *RAS*-mutant CRLM after induction systemic treatment. CtDNA analysis was performed by droplet digital PCR using blood samples collected at baseline, before and after resection. Pathologic response of CRLM was determined via the Tumour Regression Grading system.

**Findings:**

With a median follow-up of 19.6 months, the median RFS for patients with detectable (*N* = 6, [26%]) and undetectable (*N* = 17, [74%]) postoperative ctDNA was 4.8 *versus* 12.1 months, respectively. Among 21 patients with available tumour tissue, pathologic response in patients with detectable compared to undetectable postoperative ctDNA was found in one of six (17%) and 15 of 15 (100%) patients, respectively (*p* < 0.001). In univariable Cox regression analyses both postoperative detectable ctDNA (HR = 3.3, 95%CI = 1.1–9.6, *p* = 0.03) and pathologic non-response (HR = 4.6, 95%CI = 1.4–15, *p* *=* 0.01) were associated with poorer RFS and were strongly correlated (*r* = 0.88, *p* < 0.001). After adjusting for clinical characteristics in pairwise multivariable analyses, postoperative ctDNA status remained associated with RFS.

**Interpretation:**

The detection of postoperative ctDNA after secondary resection of CRLM is a promising prognostic factor for RFS and appeared to be highly correlated with pathologic response.

**Research in context**

**Evidence before this study**

Recurrence rates after resection of colorectal liver metastases (CRLM) are high and caused by micro-metastases left *in situ* after resection. Currently available follow-up methods have limited accuracy for detecting this minimal residual disease (MRD). Studies in patients with stage I-III colorectal cancer demonstrated that postoperative circulating tumour DNA (ctDNA) is a strong independent prognostic biomarker for MRD and recurrence-free survival. Studies investigating postoperative ctDNA in stage IV disease are limited and mostly concern heterogeneous patient groups with both hepatic and extrahepatic disease and varying use of induction systemic treatment.

**Added value of this study**

This is a proof of concept study reporting on the prognostic value of ctDNA in an upfront carefully selected homogeneous population of patients with *RAS* mutant initially unresectable CRLM. In addition, this is the first study to analyse the association of postoperative ctDNA detection with pathologic response in patients with metastatic CRC. CtDNA analysis was performed using the relatively fast, inexpensive, and highly sensitive droplet digital PCR to facilitate translation to future clinical practice.

**Implications of all the available evidence**

The results of this study offer a perspective on the clinical relevance of the assessment of postoperative ctDNA in CRLM patients with a high risk of recurrence. Liquid biopsy ctDNA offers the possibility for longitudinal follow-up, whereas pathologic response can only be assessed after resection. This offers opportunities for the personalisation of postoperative disease management in this common subgroup of patients with metastatic CRC, *e.g.* by intensifying follow-up or providing adjuvant treatment.

Alt-text: Unlabelled box

## 1 Introduction

The liver is the primary metastatic site of colorectal cancer (CRC). In patients with metastatic CRC, 70 to 80% have liver metastases [1]. In patients with liver-limited colorectal cancer liver metastases (CRLM), resection offers the only chance for cure or long-term survival [1]. Approximately 20% of patients present with upfront resectable CRLM (primary resectable), and 20–40% of patients with initially unresectable CRLM may convert to resectable disease upon downsizing by induction systemic treatment (secondary resectable) [2]. Nevertheless, reported 3-year recurrence rates for primary and secondary resectable CRLM are up to 60% [3,4] and 80% [4,5], respectively. The majority of recurrences occur within the first two years following resection [4]. Furthermore, over half of the CRLM patients die within five years following resection [4,6]. Pathologic response [7] and early recurrence [8] have been correlated with overall survival in patients with CRLM.

Recurrences are considered to be caused by minimal residual disease (MRD) consisting of micro-metastases left *in situ*. Currently, available follow-up methods like serum carcinogenic embryonic antigen (CEA) and cross-sectional clinical imaging such as CT- or PET-scans have limited accuracy for detecting MRD due to low sensitivity and specificity [9]. While magnetic resonance imaging (MRI) shows a higher sensitivity compared to CT-scan for detecting small and disappearing metastases in the liver after systemic therapy [10], CT-scan has a higher overall diagnostic accuracy for detecting extrahepatic disease and has clear logistical advantages compared to whole-body MRI. Determining MRD by detecting cell-free circulating tumour DNA (ctDNA) after local treatment of CRLM may offer an alternative approach with important prognostic and therapeutic implications.

Liquid biopsy-derived ctDNA represents a minimally invasive, cancer-specific biomarker with great potential to improve diagnosis and to better determine prognosis, predict drug responsiveness and monitor treatment response [11], [12], [13]. Its short half-life makes ctDNA a dynamic marker indicating the presence of cancer cells and may detect evidence of tumour response or recurrences earlier than imaging and clinical parameters [14,15]. In addition, ctDNA has the potential to provide information about the genomic changes of the tumour [16]. In patients with stage I-III CRC, postoperative ctDNA is a strong independent prognostic biomarker for MRD and recurrence-free survival (RFS) [17], [18], [19]. These data suggest that ctDNA may be a potential marker for selecting early-stage CRC patients for adjuvant systemic therapy [15,[20], [21], [22], [23], [24]]. Compared to other tumour types, patients with metastatic CRC show among the highest levels of detectable ctDNA [24,25]. In unselected patients with metastatic CRC, multiple studies have shown that detectable postoperative ctDNA is also strongly correlated with recurrence rate [26], [27], [28], [29]. However, most of these results were obtained from studies with a small and heterogeneous study population, with limited data on patients with liver-only metastatic disease. Besides, there are no studies involving patients with metastatic CRC that correlated ctDNA results with pathologic response.

The present study makes use of a well-defined selected group of patients participating in a prospective randomised study and aims to determine the prognostic value of postoperative ctDNA for detection of MRD and RFS in patients with CRLM after induction systemic therapy and complete resection of liver metastases. Secondly, the association between postoperative ctDNA detection and pathologic tumour response in liver metastases was evaluated.

## 2 Methods

### 2.1 Patient selection

Patients were selected from the ongoing CAIRO5 randomised phase 3 trial of the Dutch Colorectal Cancer Group (DCCG), in which the currently most effective first-line systemic regimens of chemotherapy plus targeted therapy are being compared in patients with initially unresectable CRLM (registration number: NCT02162563). A total of 564 patients are planned to be enrolled in the CAIRO5 clinical trial based on statistical assumptions previously described [30]. CRLM are deemed initially unresectable after assessment following predefined baseline resectability criteria considering R0-resection cannot be achieved in one procedure with one surgical intervention only. Patients are stratified for *RAS* and *BRAF V600E* mutation status and sidedness of primary tumour. Mutation analyses were performed on DNA isolated from the primary tumour for most patients because tissue from metastases was rarely available (91% *versus* 9%, respectively). Patients are evaluated every two months by an expert panel of liver surgeons and abdominal radiologists for the possibility of local treatment of CRLM following current practice [31]. Patients in whom local treatment of CRLM is achieved continue postoperatively with the preoperative systemic regimen but without the targeted agent for a total duration of pre- and postoperative treatment of six months. After patients signed informed consent, formalin-fixed paraffin-embedded (FFPE) tumour tissue was collected prior to treatment for translational research. In addition, blood samples were collected longitudinally every two months until resection and every three months after resection. For the current observational translational research subgroup analysis patients were selected who were randomised between the start of the study (June 2014) and August 2018, with *RAS* mutated tumours treated with bevacizumab plus either doublet or triplet chemotherapy, complete (R0/R1) resection of the primary tumour and liver metastases (resection and/or local ablation), and available baseline, pre- and postoperative liquid biopsies. Follow-up was recorded until May 2020. ctDNA analyses were performed on the subset of patients with *RAS* hotspot mutations, which can be analysed using the relatively fast, inexpensive and highly sensitive ddPCR test. Patients with a first postoperative liquid biopsy drawn after starting adjuvant systemic therapy were excluded to avoid the confounding effect of chemotherapy. After completing systemic treatment, follow-up was performed according to the standard of care, including a three-monthly clinical review, six-monthly serum CEA, and CT imaging.

### 2.2 Ethics

The medical ethical committee of the Amsterdam Medical Center approved the CAIRO5 study under reference number METC 2014_008, NL47650.018.14, and all patients signed written informed consent for study participation as well as liquid biopsy and tumour tissue collection for translational research.

### 2.3 Clinicopathological data

Baseline clinicopathological patient characteristics were prospectively collected, such as age, sex, characteristics of the primary tumour (sidedness of the tumour, type of *RAS* mutation), time to metastases (with metachronous disease defined as a disease-free interval of more than six months after diagnosis of the primary tumour [32]), size and number of metastases, serum CEA levels, clinical risk score (CRS) [33] (low risk 0–2 points and high risk 3–5 points), chemotherapy regimen (doublet or triplet), number of cycles and documented radiologic response according to the RECIST 1.1 criteria, type of local therapies for CRLM, and R-status of resections (R0 or R1).

Pathologic response assessment was done by evaluating hematoxylin- and eosin-stained slides by an independent pathologist blinded for ctDNA outcomes. Pathologic response was scored according to the tumour Regression Grading (TRG) [34]. TRG was graded from 1 to 5, with TRG 4 and 5 indicating no or minor pathological response.

Previous studies have shown that early recurrence after resection of CRLM, defined as recurrence within six to eight months, correlates with prognosis [8,35,36]. Therefore, we defined early recurrence as occurring within eight months of local treatment of CRLM. RFS was calculated from the date of hepatic resection until documented progression or censored on the last clinical visit date. In the case of a two-stage hepatic resection, RFS was calculated from the last surgical procedure.

### 2.4 Cell-free DNA isolation and quantification

Prior to systemic treatment (baseline), preoperatively, a maximum of 100 days postoperatively, and during follow-up, 10 ml of blood was collected using a cell-stabilising BCT® tube (Streck, La Vista, USA) at the medical centre of inclusion. For analyses, all liquid biopsies were shipped to the Clinical Chemistry laboratory at the Netherlands Cancer Institute (Amsterdam, the Netherlands). Cell-free plasma was collected in a two-step centrifugation process; 10 min at 1.700 g followed by 10 min at 20.000 g before storage at −80 °C. Cell-free DNA (cfDNA) was isolated using the QIAsymphony (Qiagen, Germany) with an elution volume set to 60 µl. The concentration of the cfDNA was measured using the Qubit™ dsDNA High-Sensitivity Assay (TFS, Waltham, USA) and ranged from 0.12 to 60.4 ng/µl.

### 2.5 Cell-free DNA *RAS* mutation analyses

*KRAS* and *NRAS* mutation analyses using extracted cfDNA from plasma were performed by droplet digital PCR (ddPCR) (Bio-Rad, Hercules, USA). For these analyses, the ddPR™ KRAS G12/G13 (#1863506), ddPCR™ KRAS Q61 (#12001626), ddPCR™ KRAS A146 (#10049550) and the ddPCR™ NRAS Q61 (#12001006) Screening Kits were used according to the manufacturer's instruction making use of 1 µl multiplex assay, 11 µl ddPCR supermix for probes (no dUTP), 9 µl sample and 1 µl H2O. When necessary, samples were diluted to 2 ng/µl. All measurements were performed in duplicate and included a blank (nuclease-free water) and an in-house positive control. Data were analysed using the QuantaSoft™ software version 1.6.6 (Bio-Rad, Hercules, USA). Individual wells with less than 10.000 total events (droplets) were excluded from the analysis, and all results were corrected based on a predefined false-positive rate, based on 60-fold analyses of commercial reference wildtype DNA (Promega; Fitchburg, WI, USA) [37].

### 2.6 Statistics

Patient and tumour characteristics were summarised as frequency counts and percentages, or as medians and range. Differences between groups were analysed using Pearson's chi-square test and Fisher exact test, as appropriate. Survival data were analysed using the Kaplan-Meier method, and survival curves were compared using the log-rank test. Cox proportional hazards regression analysis was performed to analyse prognostic factors for RFS. Hazard ratios (HRs) and corresponding 95% confidence intervals (95% CI) were estimated. Given the small sample size and the limited number of events available, a maximum of two variables was introduced in multivariable analyses. Given the strong association between ctDNA and pathologic response, they were not analysed together in the same multivariable model. A multivariable Cox regression analysis including more than one covariate together with postoperative ctDNA was performed as sensitivity analysis. Spearman's correlation coefficient was estimated to evaluate the association between pathologic response and postoperative ctDNA status. Analyses were performed using SPSS software version 25 (IBM, New York, USA).

### 2.7 Role of the funding source

This study was supported by the Dutch Cancer Society (Grant No. 10438) and by a scientific grant from Amgen, The Netherlands. The funders had no role in the design, conduct and submission of the study, nor the decision to submit the manuscript for publication. All authors had full access to all the data in the study and accepted the responsibility to submit for publication.

## 3 Results

### 3.1 Patient characteristics

**Fig. 1:**
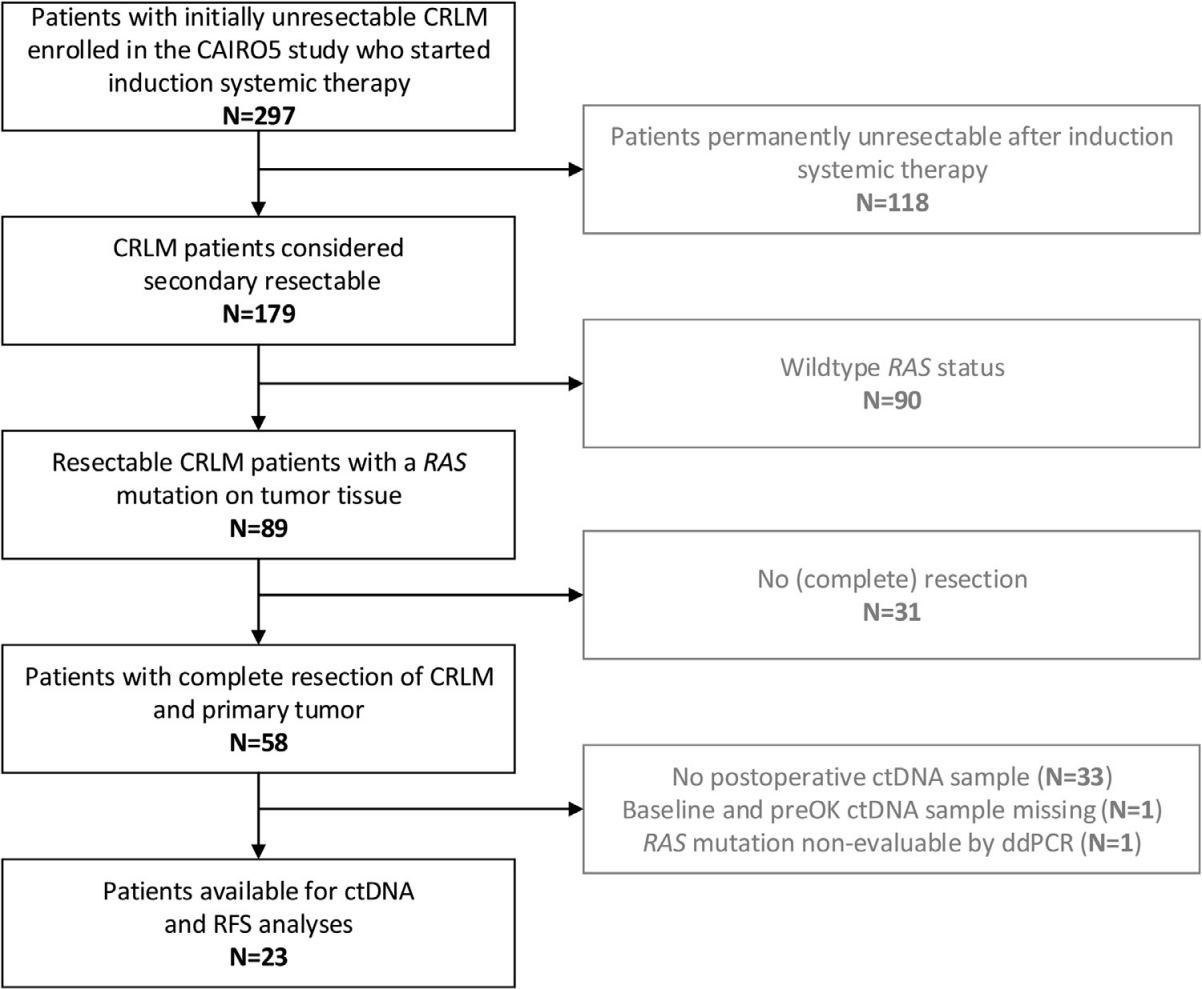
Flowchart of patient selection.

Patient selection and study overview are presented in Fig. 1. Between November 2014 and August 2018, 297 patients with initially unresectable CRLM were enrolled in the CAIRO5 study. According to tumour tissue analyses, fifty-nine patients carried a *RAS* mutation and achieved a confirmed complete resection of liver metastases and primary tumour after systemic induction therapy. After exclusion of patients with unavailable preoperative and/or postoperative liquid biopsies, a total of 23 patients, one with a *NRAS* mutation and 22 with a *KRAS* mutation, were eligible for further ctDNA and RFS analysis. The follow-up was recorded until the 20th of April 2020. The baseline patient characteristics of this cohort are displayed in Table 1 and show synchronous metastases in 19 (83%) patients, with a median number of metastases of eight (range 1–37), and 20 (87%) patients with a high CRS. Ten (44%) patients received doublet chemotherapy (FOLFOX or FOLFIRI) plus bevacizumab, and 13 (57%) patients triplet chemotherapy (FOLFOXIRI) plus bevacizumab.

**Table 1:** Summary of clinicopathological patient characteristics.

Clinical characteristics		All patients (*N* = 23)
**Age, median (range)**		63 (54–76)
**Sex, *n* (%)**		
	Male	15 (65)
	Female	8 (35)
**Tumour site, *n* (%)**		
	Left colon	17 (74)
	Right colon	6 (26)
***RAS* mutation, *n* (%)**		
	*KRAS* mutation	22 (96)
	*NRAS* mutation	1 (4)
**Source tissue mutation analysis, *n* (%)**		
	Primary tumour	21 (91)
	Liver metastases	2 (9)
**Synchronous liver metastases, *n* (%)**		
	No	4 (17)
	Yes	19 (83)
**Number of metastases, median (range)**		8 (1–37)
**Prior resection of primary tumour, *n* (%)**		
	No	11 (48)
	Yes	12 (52)
**CEA, median (range)**		9.5 (1–3469)
**Fong risk score, *n* (%)**		
	Low (0–2)	3 (13)
	High (3–5)	20 (87)
**Perioperative systemic therapy, *n* (%)**		
	Doublet chemotherapy + target therapy	10 (44)
	Triplet chemotherapy + target therapy	13 (57)
**Cycles preoperative therapy, mean (range)**		7.7 (4–13)
**Cycles postoperative therapy, mean (range)**		1.9 (0–7)
**Best response (RECIST), *n* (%)**		
	Partial response	17 (74)
	Stable disease	5 (22)
	Progression of disease	1 (4)
**Type of resection, *n* (%)**		
	1-stage	19 (83)
	2-stage	3 (13)
**R-status, *n* (%)**		
	R0	20 (83)
	R1	3 (13)
	Local ablative therapy	1 (4)
**Baseline ctDNA, *n* (%)**		
	Undetectable	2 (9)
	Detectable	18 (78)
	Missing baseline sample	3 (13)
**Histopathological response (TRG), *n* (%)**		
	Pathologic response (TRG 1–3)	16 (65)
	No pathologic response (TRG 4–5)	5 (22)
	Missing	2 (9)
**Postoperative ctDNA, days after last surgery, median (range)**		38 (1–99)

Abbreviations; CEA; carcinogenic embryonic antigen; RECIST; response evaluation criteria in solid tumours; ctDNA; circulating tumour DNA; TRG; tumour regression grade

### 3.2 Detection of ctDNA at baseline, preoperatively and postoperatively

Within the group of 23 patients, preoperative ctDNA analyses were performed on baseline blood samples in 20 patients (87%) and on preoperative blood samples in 22 patients (96%). Analyses of the postoperative liquid biopsies showed that six (26%) patients had detectable ctDNA compared to 17 (74%) patients with undetectable postoperative ctDNA. Patients with detectable *versus* undetectable postoperative ctDNA did not differ in baseline characteristics (Supplementary Table 1).

### 3.3 Association of ctDNA detection with recurrence of disease

**Fig. 2:**
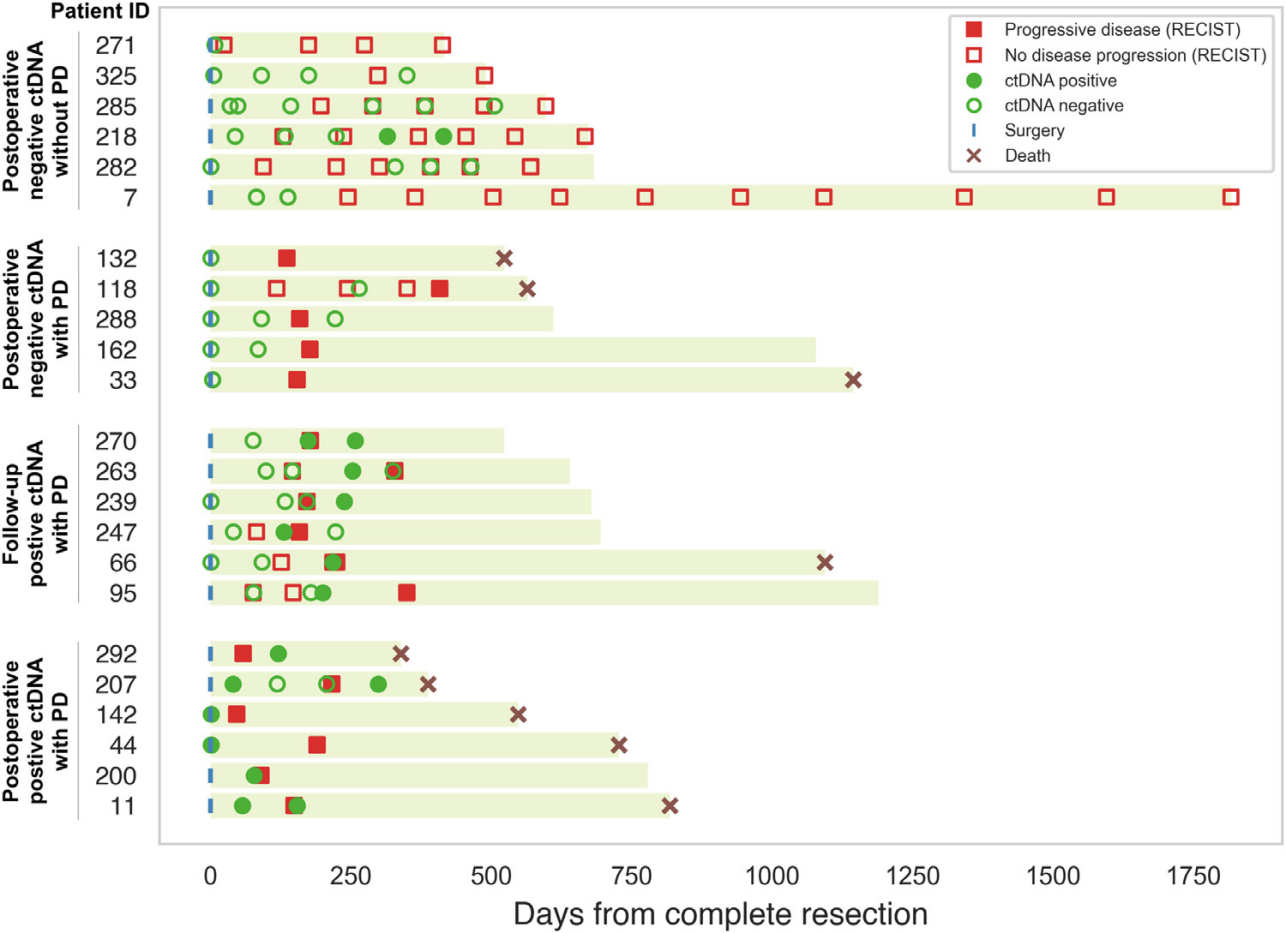
Overview of surveillance for disease recurrence in 23 patients with colorectal liver metastases (CRLM) after complete resection following induction systemic treatment. Clinical response evaluation is depicted until progression of disease (PD), where all liquid biopsy ctDNA ddPCR analysis results are showed. A distinction was made between four groups; patients with postoperative positive ctDNA with PD, patients with follow-up positive ctDNA with PD, patients with postoperative negative ctDNA with PD, and patients with postoperative negative ctDNA without PD.

**Fig. 3:**
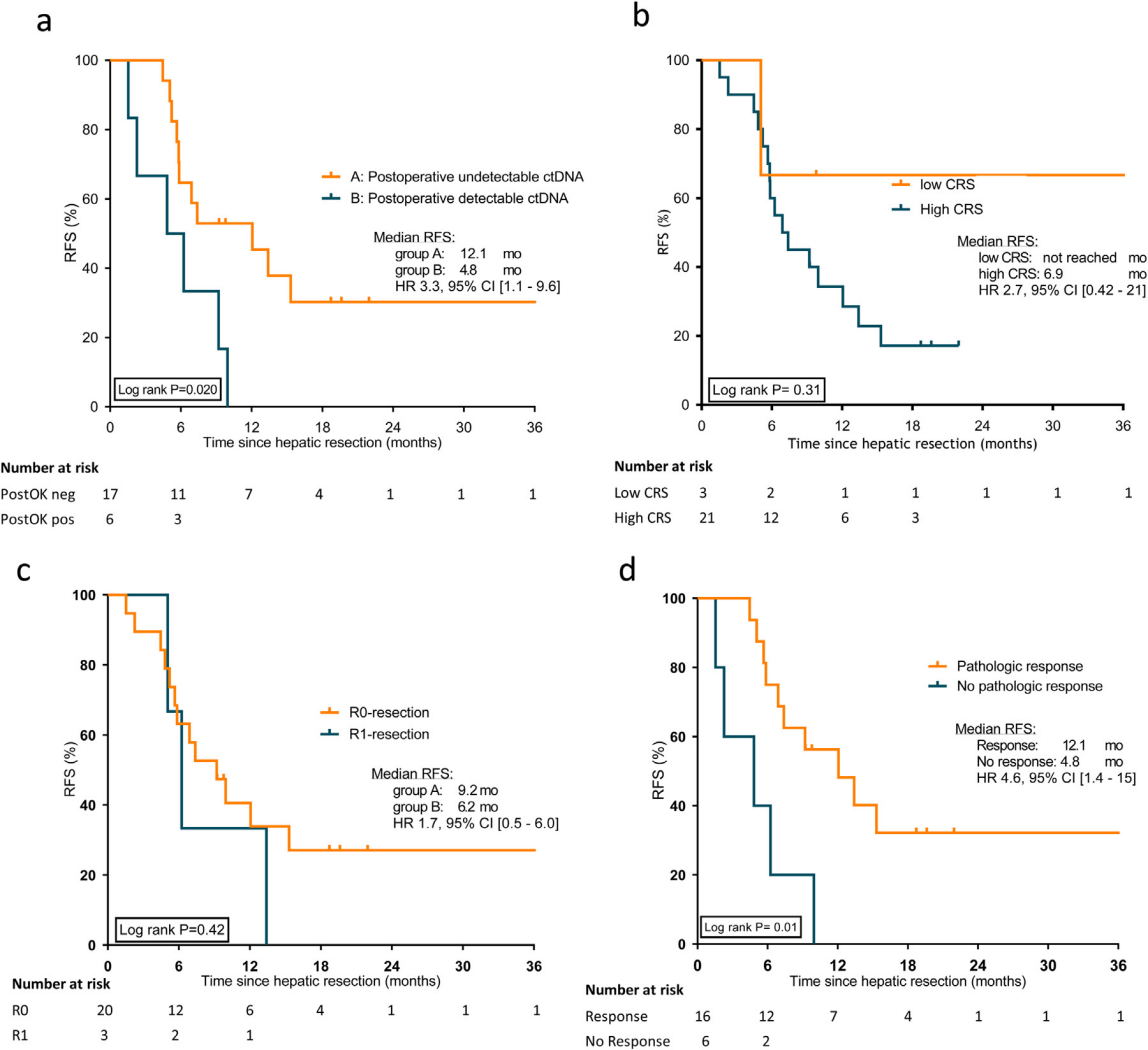
Kaplan-Meier curves showing recurrence-free survival according to: (**a**) postoperative ctDNA mutation status (undetectable *versus* detectable), (**b**) Fong clinical risk score (low *versus* high) (**c**) resection margin (R0 *versus* R1), and (**d**) pathologic response (TRG 1–3 *versus* TRG 4–5).

At a median follow-up of 19.6 months (range 1.5 – 60 months), 17 patients (74%) had recurrence of disease, with 12 patients (52%) showing early disease recurrence (≤ eight months), see Table 2. In nine patients (53%), the first recurrence occurred at an extrahepatic site. In patients with postoperatively detectable ctDNA compared to undetectable ctDNA, early disease recurrence was observed in four (67%) patients *versus* eight (47%) patients, respectively. However, this was not significant (Pearson's chi-squared test, *p* *=* 0.41, specificity 81% and sensitivity 33%). Fig. 2 presents the postoperative ctDNA status and lead-time to recurrence detected by ctDNA and imaging studies for all 23 patients. A detailed overview of both pre- and postoperative ctDNA detection per patient is presented in Supplementary Figure 1. In analysing the performance of ctDNA in the detection of MRD, we found that six patients (100%) with postoperative detectable ctDNA and 11 patients (65%) with undetectable postoperative ctDNA had a recurrence during follow-up. For a total of 15 patients, serum CEA was determined within 100 days following resection. Of patients with serum CEA levels within the normal range (*N* = 14) *versus* elevated (> 5 ng/ml) (*N* = 1), 11 (79%) and one (100%) patient developed recurrences during follow-up, respectively. Postoperative ctDNA detection was significantly associated with poorer RFS, with a median RFS for patients with postoperative undetectable *versus* detectable ctDNA of 12.1 and 4.8 months, respectively (HR 3.3, 95%CI 1.1–9.6, log-rank *p* *=* 0.03), see Fig. 3.

**Table 2:** Follow-up and recurrence-free survival for patients with postoperative undetectable and postoperative detectable ctDNA.

			All patients (*N* = 23)	Postoperative undetectable ctDNA (*N* = 17)	Postoperative detectable ctDNA (*N* = 6)
**Median follow-up, months (95% CI)**			19.6 (17.8 – 21.4)		
**Median RFS, months**			7.4	12.1	4.8
**Number of patients with recurrence, *n* (%)**			17 (74)	11 (65)	6 (100)
**Early recurrence (≤ 8 months), *n* (%)**					
	No		11 (48)	9 (53)	2 (33)
	Yes		12 (52)	8 (47)	4 (67)
**Site of recurrence**					
		Liver	8 (47)	6 (55)	2 (33)
		Extrahepatic	9 (53)	5 (45)	4 (67)
		No recurrence	6	6	–

Abbreviations; ctDNA; circulating tumour DNA; RFS; recurrence-free survival

### 3.4 Postoperative ctDNA detection and association with pathologic response

For one patient only local ablative therapy was executed, and for one patient no HE-slides were available. Therefore, pathologic response was assessed on resected tissue from liver metastases of 21 (91%) patients using Slide Score [38]. In patients with liver metastases available for pathologic response assessment, major pathologic response (TRG 1 or 2), partial (TRG 3), and no pathologic response (TRG 4 or 5) was scored in 10 (48%), six (29%), and five (24%) patients, respectively. Postoperative ctDNA status was strongly correlated with pathologic response (TRG 1–3) (Spearman's correlation, *r* = 0.88, *p* < 0.001). All patients (*N* = 15, 100%) with undetectable ctDNA had partial or major pathologic response compared to only one (17%) patient with detectable ctDNA (Pearson's Chi-squared test, *p* < 0.001).

### 3.5 Postoperative ctDNA and pathologic non-response are associated with poor RFS

Univariable survival analysis showed detectable postoperative ctDNA (HR 3.3, 95%CI 1.1–9.6, log-rank *p* = 0.03) and pathologic non-response (TRG 4–5) (HR 4.6, 95%CI 1.4–15, log-rank *p* = 0.01) to be associated with poorer RFS (see Table 3). After adjusting postoperative ctDNA for age, sex, Fong CRS, radiological response, sidedness and R-status in separate pairwise multivariable analyses, detectable postoperative ctDNA remained significantly associated with poorer RFS. The association between postoperative ctDNA and RFS remained strong in the sensitivity analysis adjusting for all the aforementioned variables simultaneously in a multivariable model (HR 4.1, 95%CI 1.19–14.47, log-rank *p* = 0.026). No indications of an association between RECIST response or non-response and pathologic response (Fisher's Exact, *p* = 0.761), detection of postoperative ctDNA (Fisher's Exact, *p* = 0.083), or recurrence of disease (Fisher's Exact, *p* = 0.217) was found.

**Table 3:** Cox regression univariable recurrence-free survival analysis by clinicopathological variables and postoperative ctDNA status.

Variable	Number patients	Event RFS		Univariable analysis		
	*n* (%)	*n*		HR	95% CI	Log-rank *P*
**Age, years**						
< 60	8 (35)	6				
> 60	15 (65)	11		1.2	0.4 – 3.1	0.78
**Sex**						
Male	15 (65)	11				
Female	8 (35)	6		0.98	0.4 – 3.7	0.98
**Sidedness primary tumour**						
Left	17 (74)	13				
Right	6 (26)	4		1.2	0.4 – 3.8	0.74
Clinical risk score*						
Low	3 (13)	1				
High	20 (87)	16		2.7	0.4 – 21	0.33
**Postoperative serum CEA**						
Normal	15 (94)	10				
Elevated (> 5 ng/ml)	1 (6)	1		0.9	0.1 – 6.8	0.90
**Resection status**						
R0-resection	19 (86)	13				
R1-resection	3 (14)	3		1.7	0.5 – 6.0	0.43
Radiological response on induction treatment						
Response	17 (74)	14				
No Response	6 (26)	3		2.5	0.7 – 8.7	0.16
**Tumour regression grade**						
Response (TRG 1–3)	16 (76)	10				
No response (TRG 4–5)	5 (24)	5		**4.6**	**1.4 – 15**	**0.01**
**Postoperative ctDNA status**						
Undetectable	17 (74)	11				
Detectable	6 (26)	6		**3.3**	**1.1 – 9.6**	**0.03**

Abbreviations; RFS; recurrence-free survival; CEA; carcinogenic embryonic antigen; TRG; tumour regression grade; ctDNA; circulating tumour DNA; *Clinical risk groups are classified according to Fong

## 4 Discussion

This study analysed the association between postoperative ctDNA and both pathologic response and RFS in patients with initially unresectable CRLM after radical resection of both CRLM and primary tumour. The results indicate that postoperative ctDNA analysis within a high-risk cohort may potentially identify patients with a higher risk of disease recurrence after secondary resection. In addition, postoperative ctDNA showed a strong association with pathologic response on systemic therapy as assessed by the tumour regression grade and is an independent prognostic factor for RFS.

Liquid biopsies are a rich source of minimal invasive biomarkers such as circulating tumour cells (CTCs) and ctDNA, which have the potential to be applied for the clinical management of patients with CRC [39]. In this study we focused on the analysis of ctDNA, considering the higher detection rate of ctDNA compared to CTCs in patients with metastatic CRC [40]. Limited data is available on the value of ctDNA in patients with CRLM [40], [41], [42]. Narayan et al. showed an association of preoperative ctDNA with overall survival in patients with upfront resectable CRLM [41]. The PRODIGE-14 METHEP-2 trial showed in initially unresectable CRLM patients that preoperative ctDNA levels correlate with R0/R1 resections and overall survival [40]. The trial of He et al. involving twenty CRLM patients, not clearly defined as initially resectable or unresectable and with approximately 50% receiving neo-adjuvant systemic therapy, demonstrated a prolonged RFS for patients with low preoperative ctDNA [42]. Further studies in patients with resected CRLM concerned heterogeneous populations in terms of CRC stage among the whole population, presence of extrahepatic metastases, first presentation and relapse of CRLM [27,28], inclusion of both radical and non-radical resections [28], primary and secondary resectable CRLM and types of local therapy [29,42].

To our knowledge, this is the first study to analyse the association of postoperative ctDNA detection and pathologic response in resected liver metastases in CRLM patients. Pathologic response is a well-known independent prognostic factor for overall survival in patients with CRLM [7] and can therefore be used as an early surrogate marker for survival. Our results show a strong association between postoperative ctDNA status and pathologic response. After adjusting for clinical characteristics, both postoperative ctDNA and pathologic response were independent prognostic factors for RFS in separately conducted pairwise multivariable analysis. The added value of ctDNA compared to pathologic response is the ability to perform serial ctDNA analyses in longitudinal follow-up, whereas pathologic response is only possible after resection. Additionally, ctDNA is analysed by a simple blood draw while pathologic response requires tumour tissue. These factors combined with the results of this study might have clinically relevant implications since ctDNA could be used as a surrogate marker for pathologic response and clinical outcome in metastatic CRC patients without available tumour tissue after systemic therapy, such as patients treated with local ablative therapy only or patients on palliative systemic therapy.

The promising monitoring and prognostic value of ctDNA have raised major interest in ctDNA driven adjuvant trials [43]. Adjuvant systemic therapy in CRLM patients has failed to show a 5 year survival benefit [6]. However, this study concerned relatively low-risk CRLM patients (with four or fewer metastases), and retrospective studies suggest that an adequate selection of patients with a high risk of recurrence could help select the patients who might benefit from adjuvant treatment [44,45]. Our results show that postoperative ctDNA status is an independent prognostic factor for RFS and might be a promising biomarker in future trials to select very high-risk CRLM patients for adjuvant trials or otherwise for individualised therapy.

Liquid biopsy ctDNA is a promising biomarker to optimize strategies for monitoring disease recurrence after resection of CRLM. Early detection of a recurrence limited to the liver might offer an opportunity for repeated local treatments with curative intent. Further studies are needed to determine if patients with detectable postoperative ctDNA have clinical benefit from intensified follow-up strategies, like more frequent evaluations or additional imaging methods such as MRI or PET-CT, resulting in better survival outcomes than the current standard of care follow-up strategies. With the additional advantage of liquid biopsies providing the ability for longitudinal monitoring of disease recurrence, having less burden to patients and lower costs than radiological imaging, ctDNA is an interesting biomarker to investigate in future prospective trials. Furthermore, combining radiologic and ctDNA assessments might also help interpret indeterminate radiological findings such as nonspecific liver or lung nodules. Currently, serum CEA is used after resection of CRLM to monitor disease recurrence. However, serum CEA has a low sensitivity and specificity, which might be explained by expression in both neoplastic and normal cells [45], [46], [47]. Liquid biopsy ctDNA was shown to perform better [41,48] with higher sensitivity compared to serum CEA, 100% *versus* 56% (*p* = 0.01) [26]. In our population with high-risk CRLM patients, we confirmed that ctDNA is a stronger prognostic marker for RFS than CEA. Secondly, in pairwise multivariable analysis with other potential clinicopathological risk factors for disease recurrence (*e.g.* CEA, CRS, R-status), we found indications that postoperative ctDNA status was an independent prognostic factor for RFS in patients with secondary resection of CRLM.

An ideal test to diagnose MRD after resection, and further tailor adjuvant systemic treatment, has high sensitivity and specificity [49]. Previously, postoperative ctDNA in metastatic CRC was shown to have high specificity but relatively low sensitivity, since a considerable number of patients with undetectable postoperative ctDNA still developed a recurrence [27], [28], [29]. Similarly, in our study investigating a homogeneous group of CRLM patients, we found a high specificity, where all patients with postoperative detectable ctDNA had a recurrence during follow-up, but lower sensitivity, since 65% of the patients with undetectable postoperative ctDNA also developed a recurrence. A possible factor contributing to our study's sensitivity is the use of ddPCR as a hotspot detection method (detection of one mutation). Our study focused on patients whose *RAS* mutation status was determined as part of the clinical diagnostic workflow, to establish their eligibility for anti-EGFR treatment. Methodologically, ddPCR-based assays for detecting ctDNA hotspot mutations have high sensitivity and are relatively cheap [37]. This ensures more widespread applicability in daily clinical practice as compared to NGS analyses of gene panels and rendered ddPCR a logical choice for detecting ctDNA in this subset of patients in the present study. Another explanation for the phenomenon of undetectable postoperative ctDNA in patients with MRD leading to recurrence might be the use of preoperative systemic therapy in all patients in our study. This could have (temporarily) reduced the proliferation and apoptosis of minimal residual tumour cells postoperatively, thereby reducing the shedding of ctDNA [49]. Also the time window from postoperative blood draw till disease recurrence might have been too long. Lastly, the site of recurrence might have an impact on ctDNA detection in the circulation [25]. Future studies should determine the optimal time window for the sampling of ctDNA after surgery. Liquid biopsy cfDNA levels after tissue damage resulting from the surgery itself can be elevated up to four weeks, which may result in masking ctDNA with false-negative outcomes. It has been recommended that a second blood sample, collected after four weeks, is analysed for patients with postoperative undetectable ctDNA [50].

Limitations of our study include the small sample size, in part caused by the exclusion of patients with missing postoperative blood samples. The challenging logistics of blood sampling for translational research are well established [40]. Also, the sample size was limited to patients with a known *RAS* mutation, present in only 40–56% of patients with metastatic CRC [51], [52], [53]. A strength of our study is the homogeneous study population relative to other studies assessing the value of postoperative ctDNA in CRLM patients [28,29,41,42]. Integrating clinical, pathological and molecular markers can help to improve and customise therapy.

In conclusion, the detection of postoperative ctDNA is a promising prognostic factor for disease recurrence and median RFS in patients after secondary resection of *RAS* mutated colorectal cancer liver-only metastases. In addition, postoperative ctDNA showed a strong association with pathologic response. Further analysis with a bigger sample size would be needed to confirm these promising findings.

## Contributors

Study concept and design: KB, IvtE, CJAP, RJAF

Financial support: GAM, CJAP, RJAF

Data collection: KB, IvtE, CM, PMDvD, AK, RJS

Statistical analyses: KB, IvtE, MLY

Data interpretation: KB, IvtE, CJAP, RJAF

Manuscript writing: KB, IvtE, CJAP, RJAF

Critical revision of the manuscript: All authors

## Data sharing statement

This translational research study makes use of patients who participate in the currently ongoing CAIRO5 clinical trial, however, does not report clinical trial data. Therefore, except for the information presented in this study no additional individual participant data is currently available. Other available documents are the CAIRO5 study protocol and informed consent form. These are accessible via: https://dccg.nl/trial/cairo5

## Declaration of Competing Interest

C.J.A.P. has an advisory role for Nordic Pharma. This funding is not related to the current research.

G.A.M. reports non-financial support from Exact Sciences, non-financial support from Sysmex, non-financial support from Sentinel CH. SpA, non-financial support from Personal Genome Diagnostics (PGDX), other from Hartwig Medical Foundation, grants from CZ (OWM Centrale Zorgverzekeraars groep Zorgverzekeraar u.a), other from Royal Philips, other from GlaxoSmithKline, other from Keosys SARL, other from Open Clinica LLC, other from Roche Diagnostics Nederland BV, other from The Hyve BV, other from Open Text, other from SURFSara BV, other from Vancis BV, other from CSC Computer Sciences BV, outside the submitted work; In addition, G.A.M. has several patents pending.

R.J.A.F. reports grants and non-financial support from Personal Genome Diagnostics, grants from MERCK BV, non-financial support from Pacific Biosciences, non-financial support from Cergentis BV, outside the submitted work; In addition, R.J.A.F. has several patents pending.

The remaining authors declare no potential conflicts of interest.

## Appendix Supplementary materials

Image, application 1

Image, application 2

Image, application 3

Image, application 4
