# Supplementary material for: Postoperative circulating tumour DNA is associated with pathologic response and recurrence-free survival after resection of colorectal cancer liver metastases
Source: eBioMedicine. 2021 Jul 29;70:103498. doi: 10.1016/j.ebiom.2021.103498 (PMC8340125; doi:10.1016/j.ebiom.2021.103498)
Supplement: Supplementary file 1 [file mmc1.docx]

**Supplementary Table 1.** Comparison of patient characteristics between patients with postoperative undetectable and postoperative detectable ctDNA

|  | | | **All patients**  **(N = 23)** | **Postoperative undetectable ctDNA**  **(N = 17)** | **Postoperative detectable ctDNA**  **(N = 6)** | **p-value*** |
| --- | --- | --- | --- | --- | --- | --- |
| **Age, median (range)** | | | 63 (54-76) | 64 | 58 | 0·76 |
| **Sex, *n* (%)** | | |  |  |  | 0·93 |
|  | Male | | 15 (65) | 11 (65) | 4 (67) |  |
|  | Female | | 8 (35) | 6 (35) | 2 (33) |  |
| **Tumour site, *n* (%)** | | |  |  |  | 0·54 |
|  | Left colon | | 17 (74) | 12 (71) | 5 (83) |  |
|  | Right colon | | 6 (26) | 5 (29) | 1 (17) |  |
| ***RAS* mutation, *n* (%)** | | |  |  |  | 0·26 |
|  | *KRAS* mutation | | 22 (96) | 17 (100) | 5 (83) |  |
|  | *NRAS* mutation | | 1 (4) | 0 | 1 (17) |  |
| **Synchronous liver metastases, *n* (%)** | | |  |  |  | 0·19 |
|  | No | | 4 (17) | 4 (24) | 0 |  |
|  | Yes | | 19 (83) | 13 (77) | 6 (100) |  |
| **Number of metastases, median (range)** | | | 7·5 (1-37) | 8·5 | 6·5 | 0·81 |
| **Prior resection of primary tumour, *n* (%)** | | |  |  |  | 0·90 |
|  | | No | 11 (46) | 8 (47) | 3 (50) |  |
|  | | Yes | 13 (54) | 9 (5530) | 3 (50) |  |
| **CEA, median (range)** | | | 10·8 (1-3469) |  |  |  |
| **Fong risk score, n (%)** | | |  |  |  | 0·74 |
|  | | Medium (2-3) | 14 (59) | 10 (59) | 4 (67) |  |
|  | | High (4-5) | 7 (41) | 7 (41) | 2 (33) |  |
| **Perioperative systemic therapy, *n* (%)** | | |  |  |  | 0·18 |
|  | | Doublet + target therapy | 10 (42) | 6 (35) | 4 (67) |  |
|  | | Triplet + target therapy | 14 (58) | 11 (65) | 2 (33) |  |
| **Cycles neo-adjuvant therapy, mean (range)** | | | 7·8 (4-13) | 7·6 | 8·7 | 0·87 |
| **Cycles adjuvant therapy, mean (range)** | | | 1·9 (0-7) | 2·4 | 0·3 | 0·39 |
| **Best response, *n* (%)** | | |  |  |  | 0·10 |
|  | Partial response | | 17 (74) | 12 (71) | 5 (83) |  |
|  | Stable disease | | 5 (22) | 5 (29) | 0 |  |
|  | Progression of disease | | 1 (4) | 0 | 1 (17) |  |
| **Type of resections, *n* (%)** | | |  |  |  | 0·80 |
|  | | 1-stage | 20 (87) | 15 (88) | 5 (83) |  |
|  | | 2-stage | 3 (13) | 2 (12) | 1 (17) |  |
| **R-status, *n* (%)** | | |  |  |  | 0·76 |
|  | | R0 | 19 (86) | 14 (88) | 5 (83) |  |
|  | | R1 | 3 (14) | 2 (12) | 1 (17) |  |
|  | | RFA/MWA | 1 | 1 | 0 |  |
| **Baseline ctDNA, *n* (%)** | | |  |  |  | 0·48 |
|  | | Undetectable | 2 (10) | 2 (14) | 0 |  |
|  | | Detectable | 18 (90) | 12 (86) | 6 (100) |  |
|  | | Missing baseline sample | 3 | 3 | 0 |  |
| **Histopathological response (TRG) , *n* (%)** | | |  |  |  | < 0·001 |
|  | | Pathologic response (TRG 1-3) | 16 (76) | 15 (100) | 1 (17) |  |
|  | | No pathologic response (TRG 4-5) | 5 (24) | 0 (0) | 5 (83) |  |
|  | | Missing | 2 | 2 | - |  |

*Abbreviations: ctDNA: circulating tumour DNA, CEA: carcinogenic embryonic antigen, RECIST: response evaluation criteria in solid tumours, TRG: tumour regression grade. *Categorical variables were compared with the Pearson’s chi-square test and continuous variables were compared using the Mann-Whitney U test.*
