# Supplementary material for: Postoperative circulating tumour DNA is associated with pathologic response and recurrence-free survival after resection of colorectal cancer liver metastases
Source: eBioMedicine. 2021 Jul 29;70:103498. doi: 10.1016/j.ebiom.2021.103498 (PMC8340125; doi:10.1016/j.ebiom.2021.103498)
Supplement: Supplementary file 2 [file mmc2.docx]

**Caption for supplementary material**

**Supplementary Table 1.** Comparison of patient characteristics between patients with postoperative undetectable and postoperative detectable ctDNA.

**Supplementary Figure 1.** Individual plots of all 23 patients showing ctDNA dynamics in the bottom panel by depicting the mutant allele frequencies (MAF) of the identified somatic *RAS* mutation. Detected mutations are shown in red, whereas undetected mutations are shown in green. Following the same x-axis, the top panel provides information about the treatment, surgeries, and radiological assessments of the patient.
