# Supplementary material for: Postoperative circulating tumour DNA is associated with pathologic response and recurrence-free survival after resection of colorectal cancer liver metastases
Source: eBioMedicine. 2021 Jul 29;70:103498. doi: 10.1016/j.ebiom.2021.103498 (PMC8340125; doi:10.1016/j.ebiom.2021.103498)
Supplement: Supplementary file 4 [file mmc4.pdf]

Patient 7

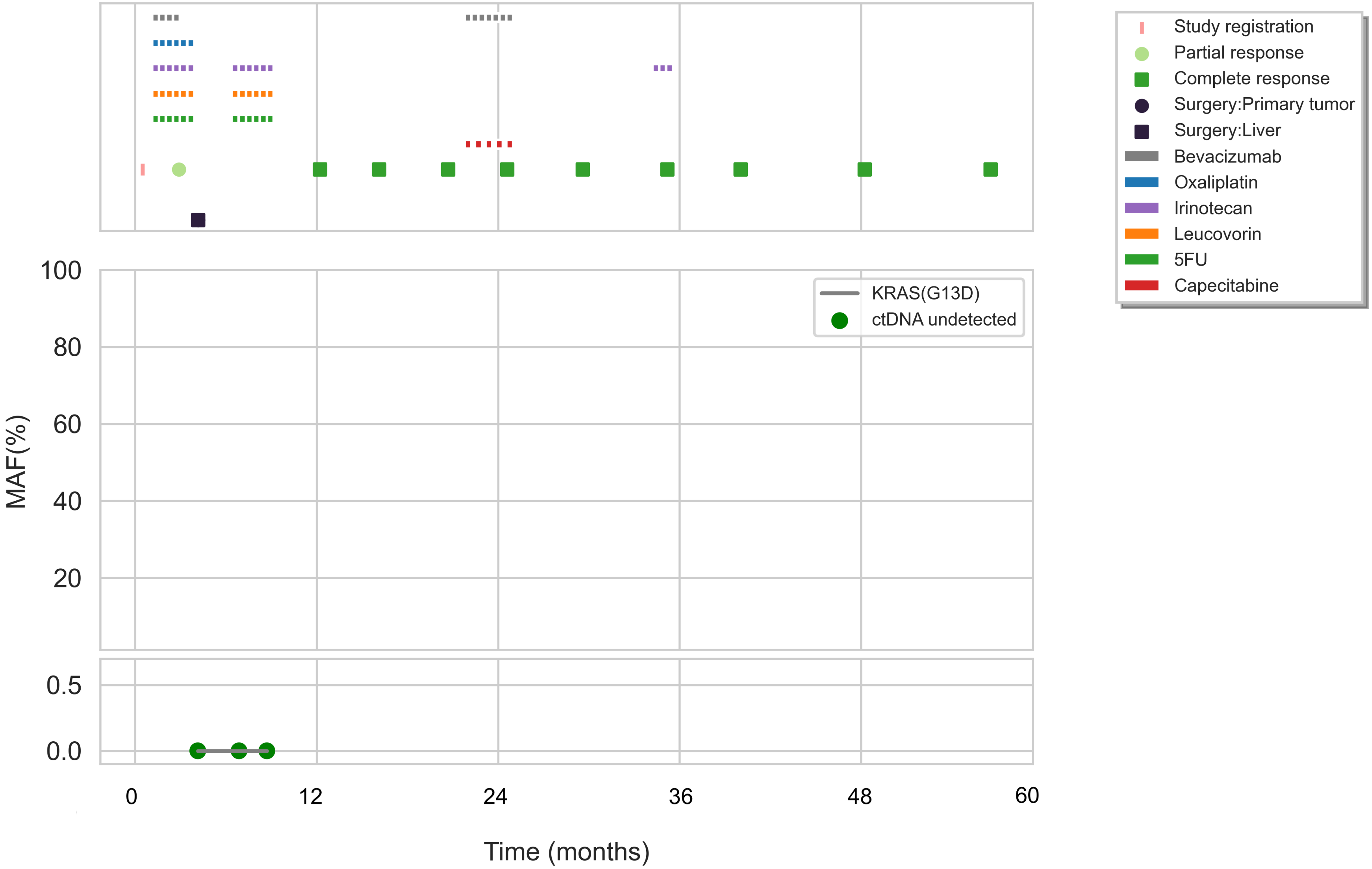

Study registration
Partial response
Complete response
Surgery:Primary tumor
Surgery:Liver
Bevacizumab
Oxaliplatin
Irinotecan
Leucovorin
5FU
Capecitabine
KRAS(G13D)
ctDNA undetected
MAF(%)
100
80
60
40
20
0.5
0.0
0
12
24
36
48
60
Time (months)

Patient 11

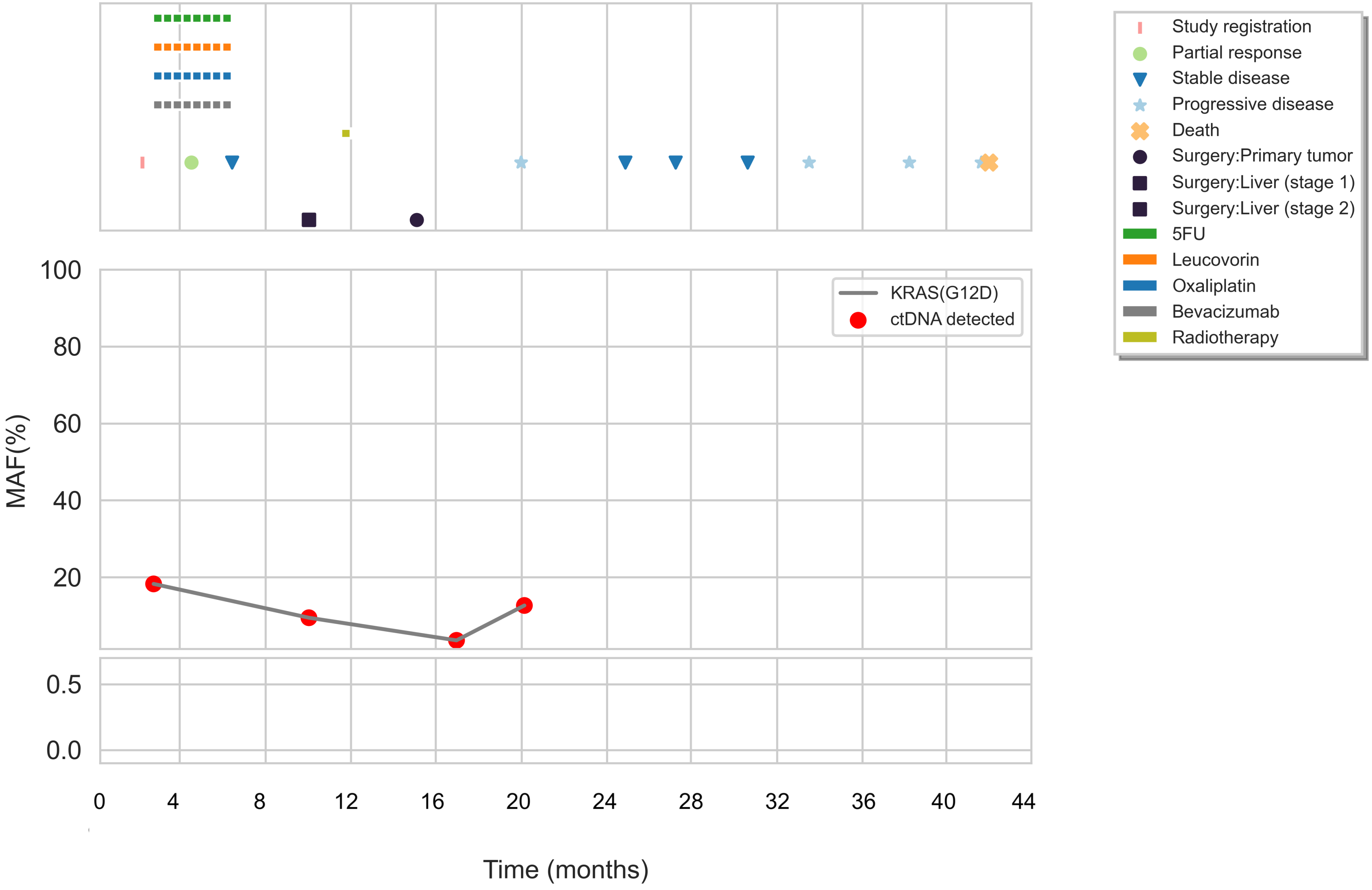

Study registration
Partial response
Stable disease
Progressive disease
Death
Surgery:Primary tumor
Surgery:Liver (stage 1)
Surgery:Liver (stage 2)
5FU
Leucovorin
Oxaliplatin
Bevacizumab
Radiotherapy
KRAS(G12D)
ctDNA detected
MAF(%)
100
80
60
40
20
0.5
0.0
0
4
8
12
16
20
24
28
32
36
40
44
Time (months)

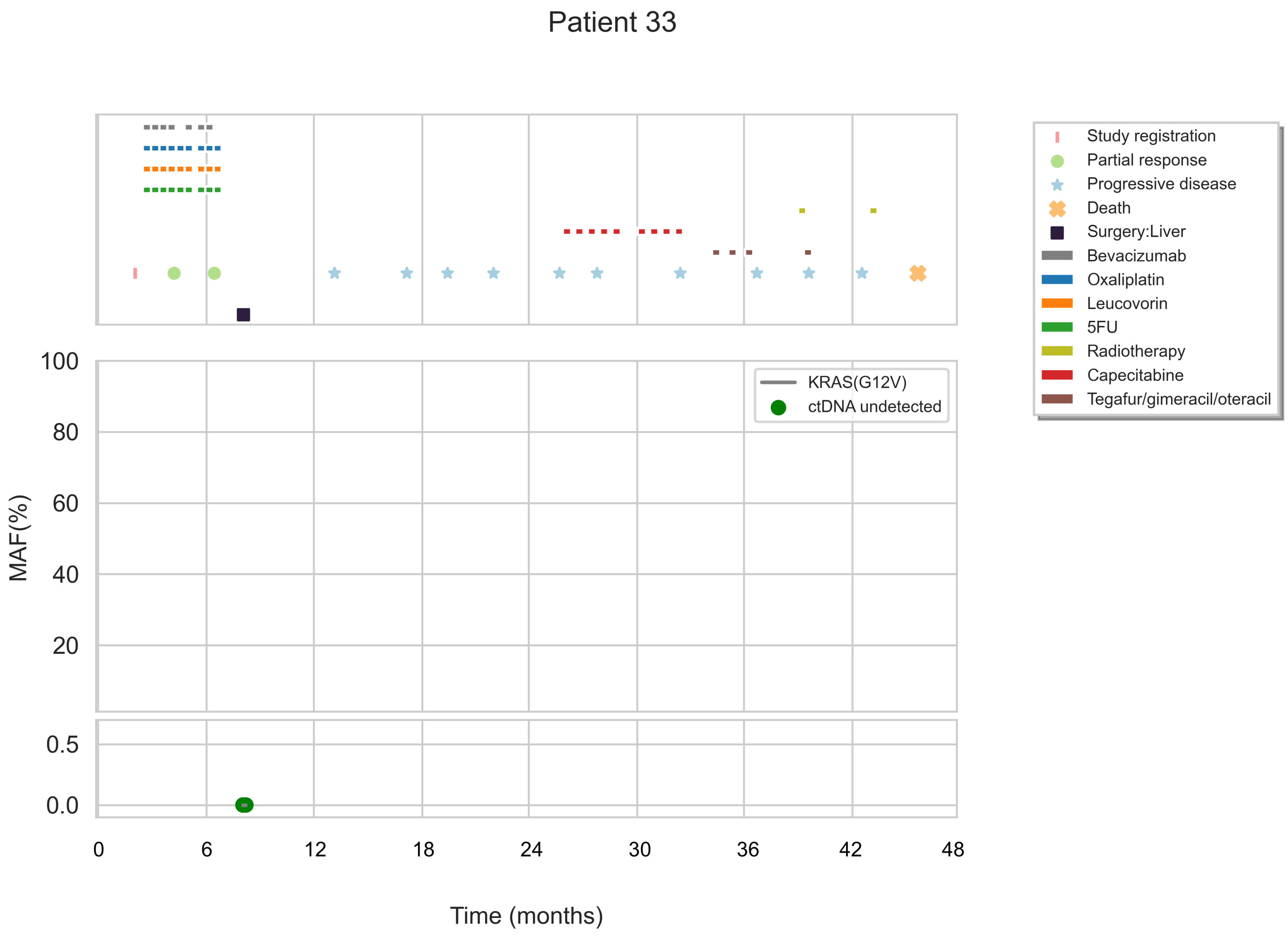

Patient 33
MAF(%)
100
80
60
40
20
0.5
0.0
0
6
12
18
24
30
36
42
48
Time (months)
KRAS(G12V)
ctDNA undetected
Study registration
Partial response
Progressive disease
Death
Surgery:Liver
Bevacizumab
Oxaliplatin
Leucovorin
5FU
Radiotherapy
Capecitabine
Tegafur/gimeracil/oteracil

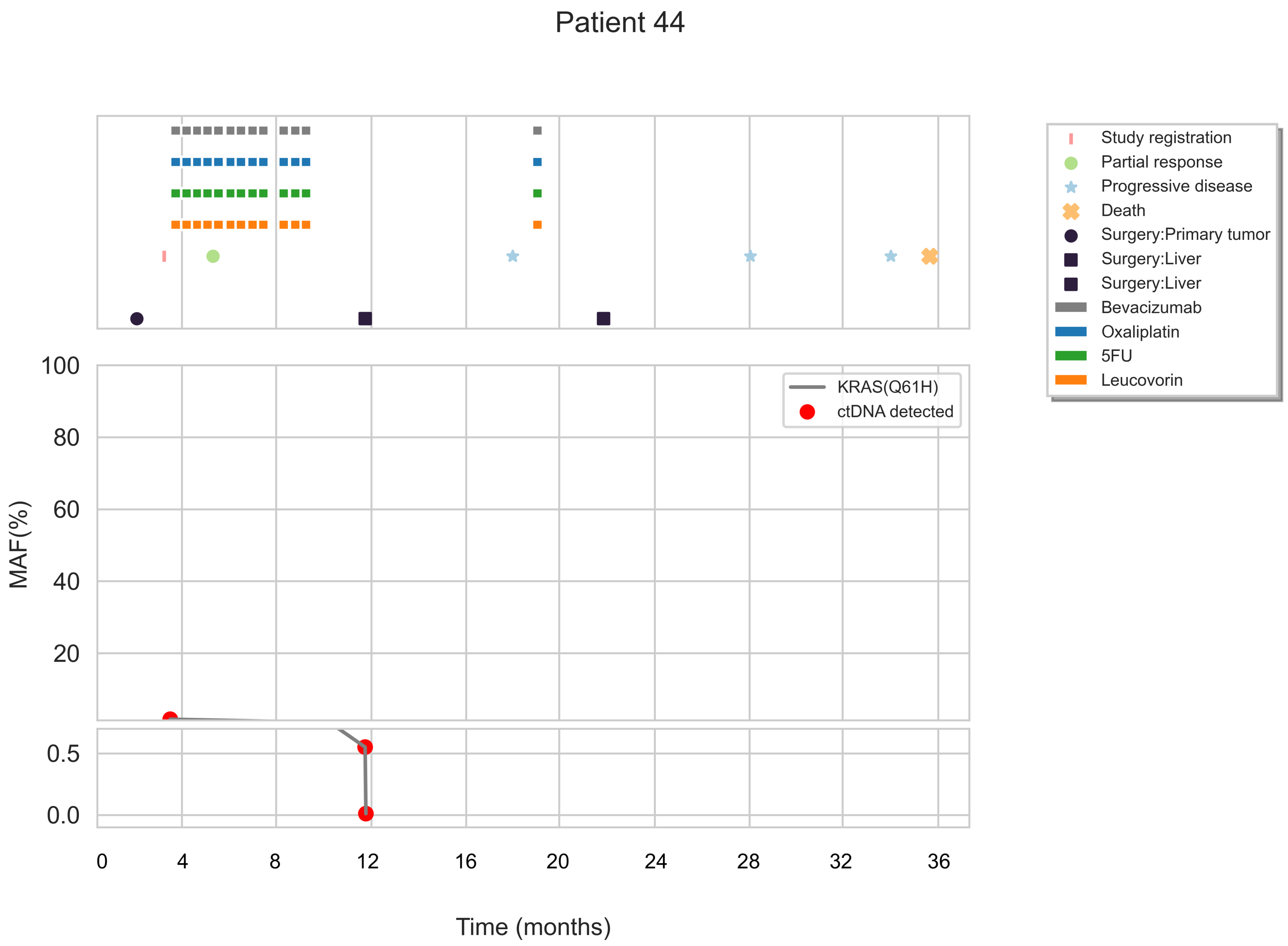
Patient 44
Study registration
Partial response
Progressive disease
Death
Surgery:Primary tumor
Surgery:Liver
Surgery:Liver
Bevacizumab
Oxaliplatin
5FU
Leucovorin
KRAS(Q61H)
ctDNA detected
MAF(%)
100
80
60
40
20
0.5
0.0
0
4
8
12
16
20
24
28
32
36
Time (months)

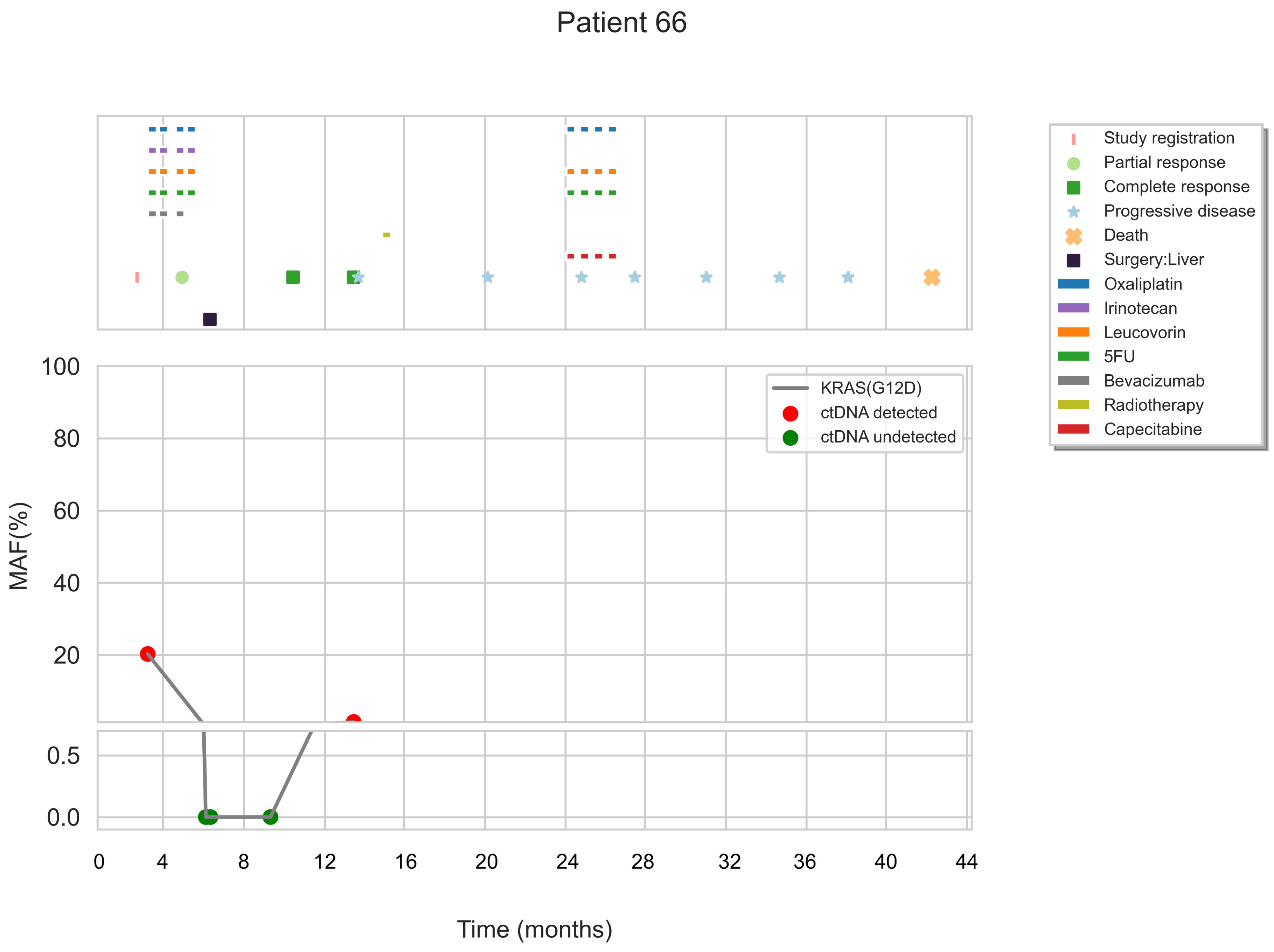

Patient 66
Study registration
Partial response
Complete response
Progressive disease
Death
Surgery:Liver
Oxaliplatin
Irinotecan
Leucovorin
5FU
Bevacizumab
Radiotherapy
Capecitabine
KRAS(G12D)
ctDNA detected
ctDNA undetected
MAF(%)
100
80
60
40
20
0.5
0.0
0
4
8
12
16
20
24
28
32
36
40
44
Time (months)

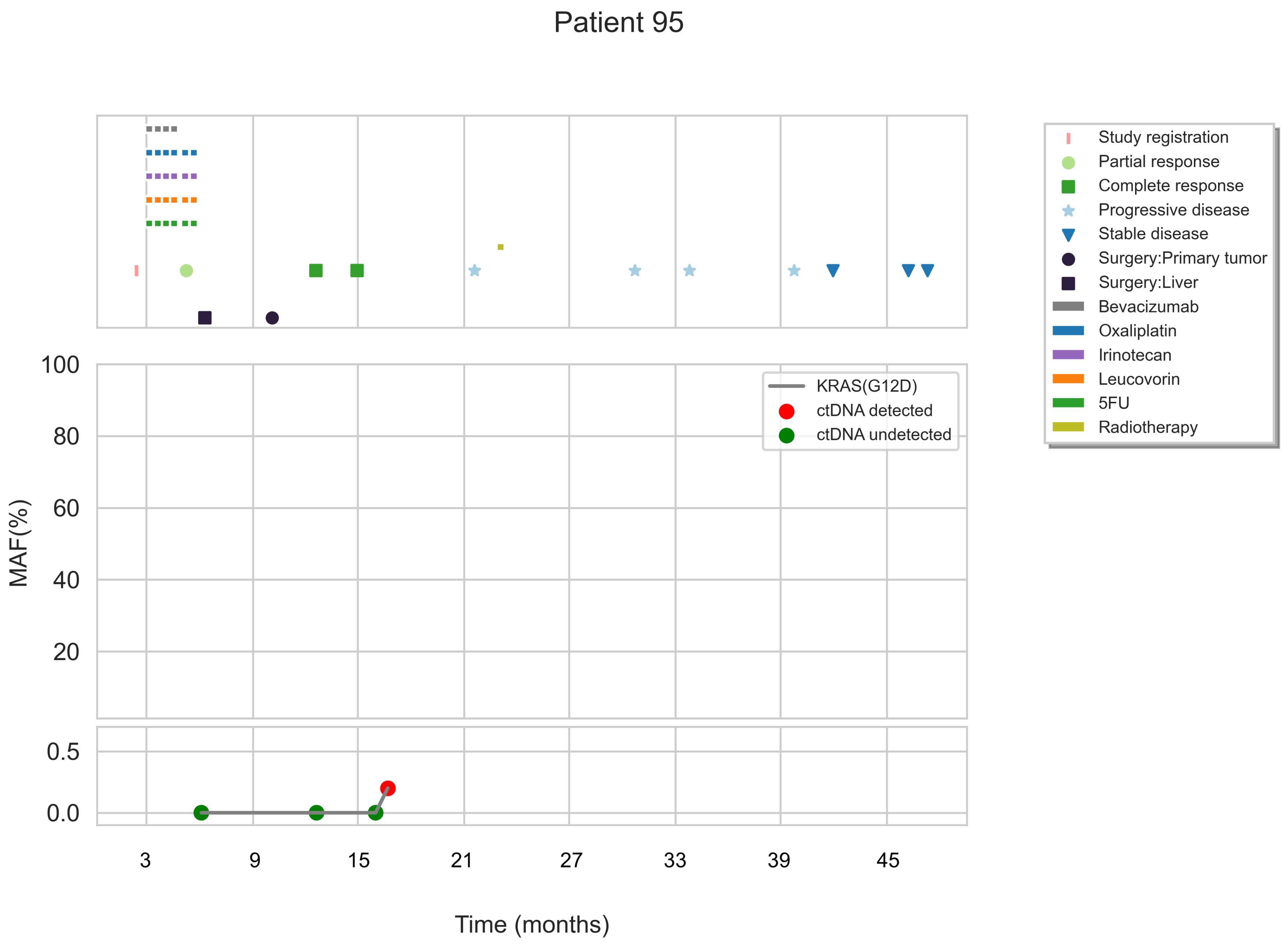

Patient 95
Study registration
Partial response
Complete response
Progressive disease
Stable disease
Surgery:Primary tumor
Surgery:Liver
Bevacizumab
Oxaliplatin
Irinotecan
Leucovorin
5FU
Radiotherapy
KRAS(G12D)
ctDNA detected
ctDNA undetected
MAF(%)
100
80
60
40
20
0.5
0.0
3
9
15
21
27
33
39
45
Time (months)

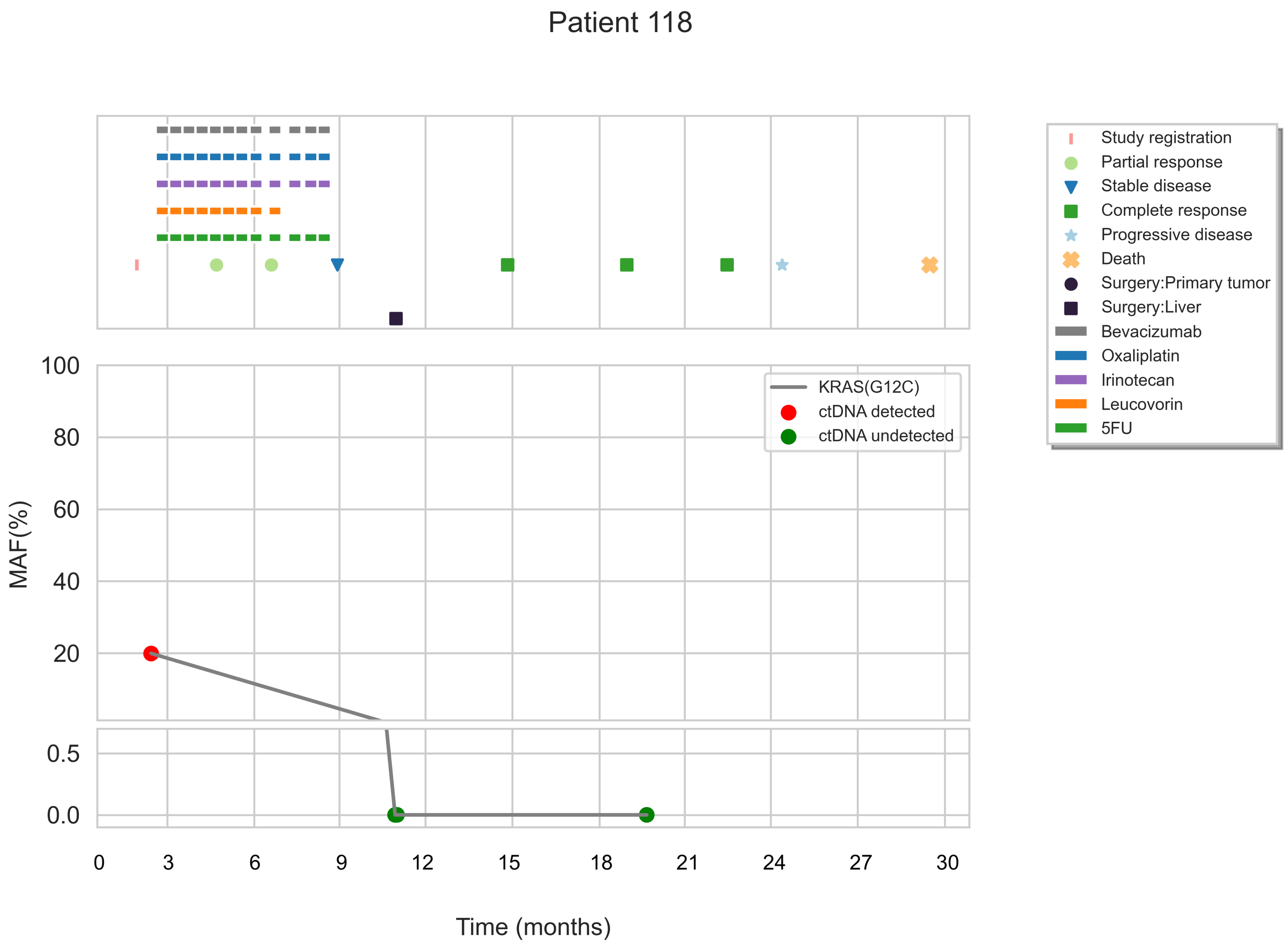

Patient 118
Study registration
Partial response
Stable disease
Complete response
Progressive disease
Death
Surgery:Primary tumor
Surgery:Liver
Bevacizumab
Oxaliplatin
Irinotecan
Leucovorin
5FU
KRAS(G12C)
ctDNA detected
ctDNA undetected
MAF(%)
100
80
60
40
20
0.5
0.0
0
3
6
9
12
15
18
21
24
27
30
Time (months)

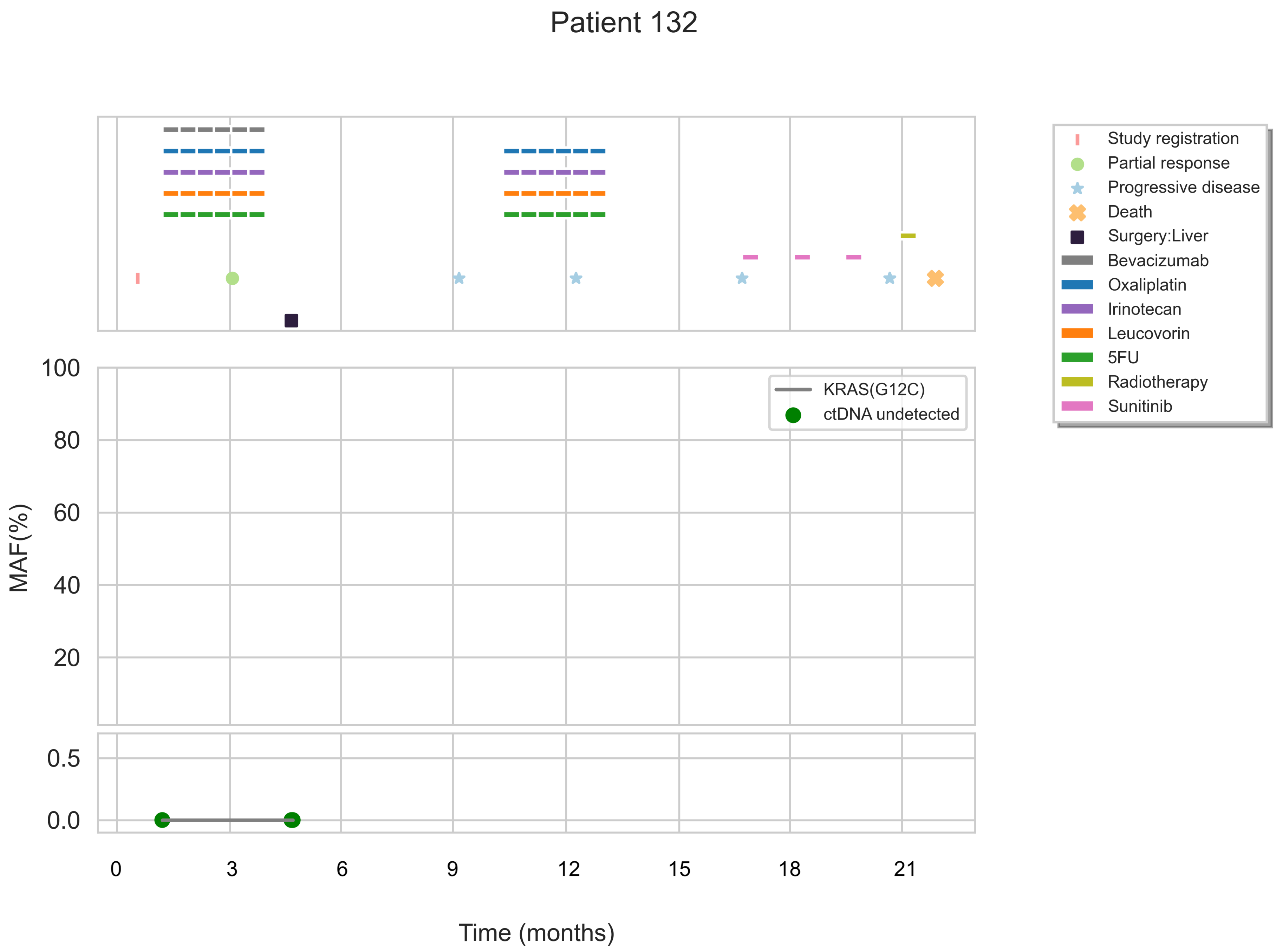
Patient 132
Study registration
Partial response
Progressive disease
Death
Surgery:Liver
Bevacizumab
Oxaliplatin
Irinotecan
Leucovorin
5FU
Radiotherapy
Sunitinib
KRAS(G12C)
ctDNA undetected
MAF(%)
100
80
60
40
20
0.5
0.0
0
3
6
9
12
15
18
21
Time (months)

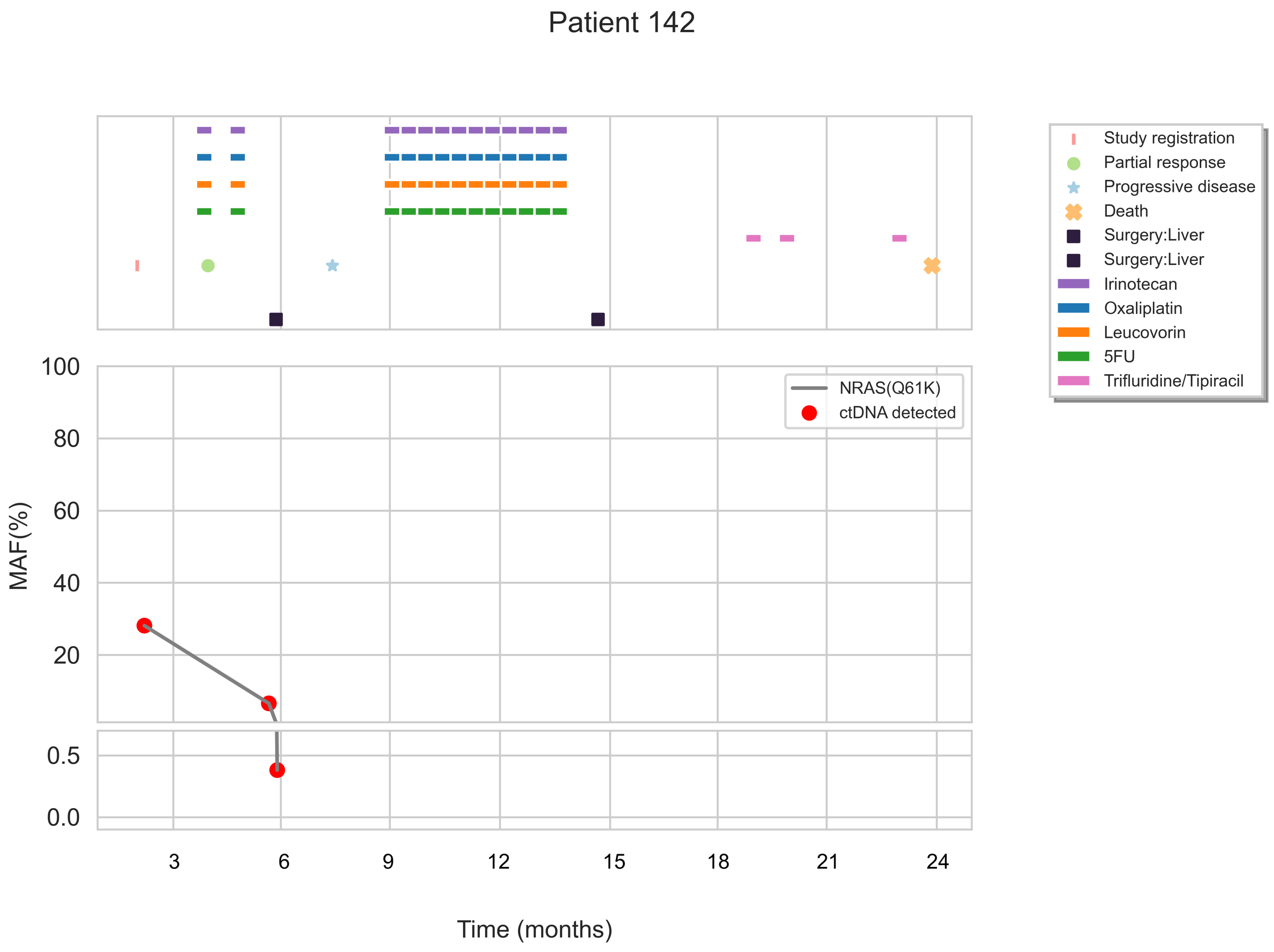

Patient 142
Study registration
Partial response
Progressive disease
Death
Surgery:Liver
Surgery:Liver
Irinotecan
Oxaliplatin
Leucovorin
5FU
Trifluridine/Tipiracil
NRAS(Q61K)
ctDNA detected
MAF(%)
100
80
60
40
20
0.5
0.0
3
6
9
12
15
18
21
24
Time (months)

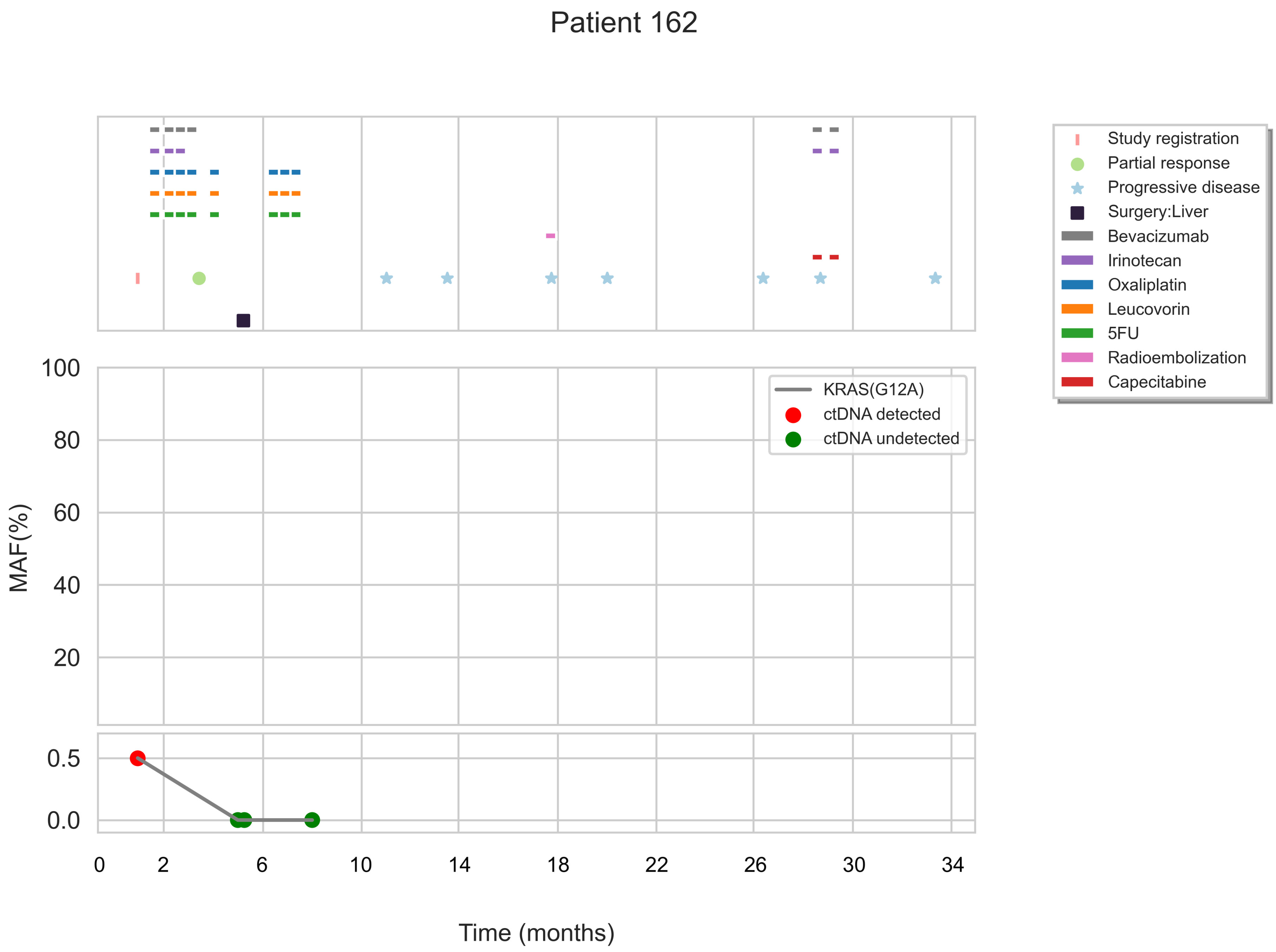
Patient 162
Study registration
Partial response
Progressive disease
Surgery:Liver
Bevacizumab
Irinotecan
Oxaliplatin
Leucovorin
5FU
Radioembolization
Capecitabine
KRAS(G12A)
ctDNA detected
ctDNA undetected
MAF(%)
100
80
60
40
20
0.5
0.0
0
2
6
10
14
18
22
26
30
34
Time (months)

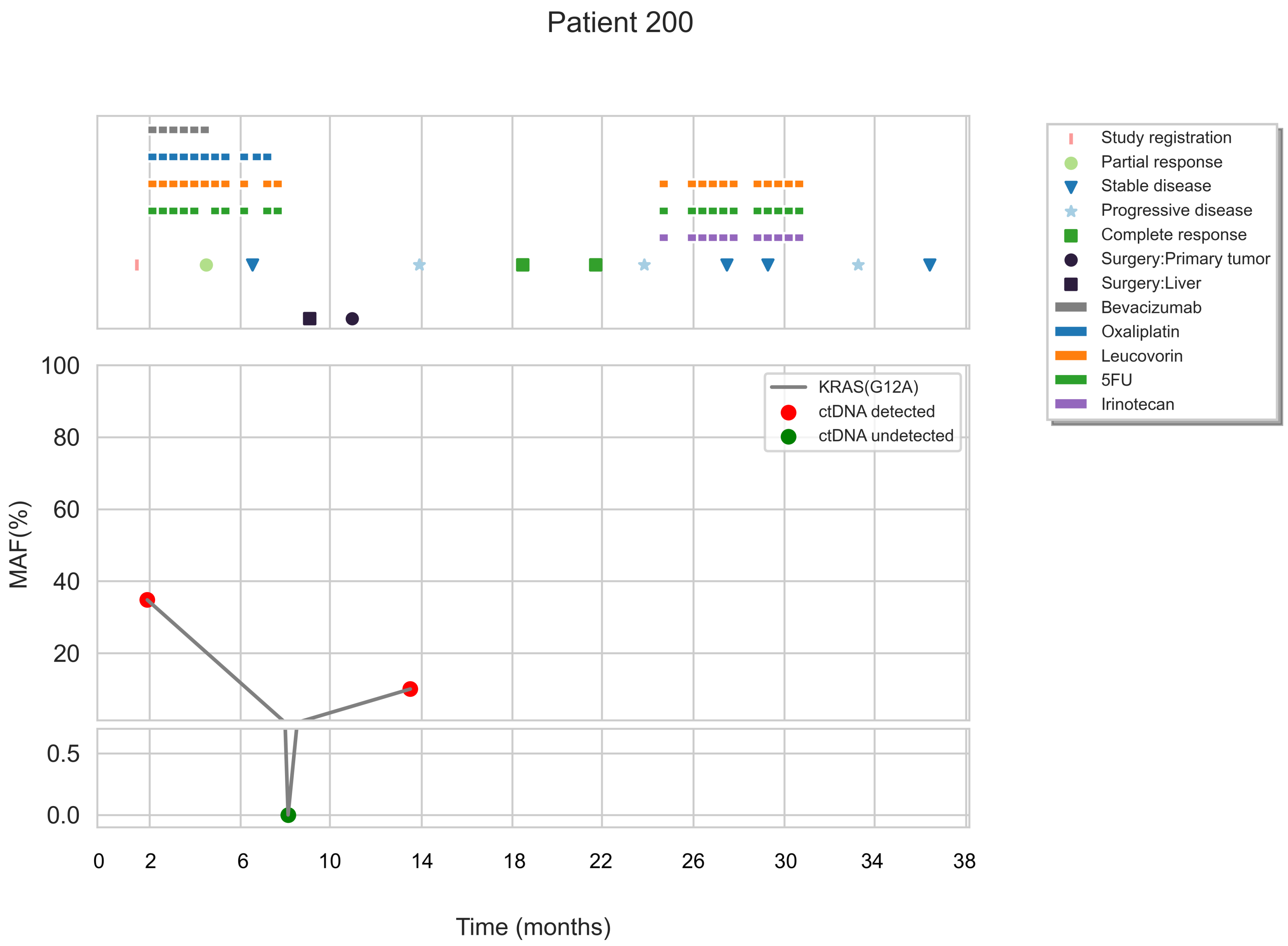

Patient 200
Study registration
Partial response
Stable disease
Progressive disease
Complete response
Surgery:Primary tumor
Surgery:Liver
Bevacizumab
Oxaliplatin
Leucovorin
5FU
Irinotecan
KRAS(G12A)
ctDNA detected
ctDNA undetected
MAF(%)
100
80
60
40
20
0.5
0.0
0
2
6
10
14
18
22
26
30
34
38
Time (months)

Patient 207

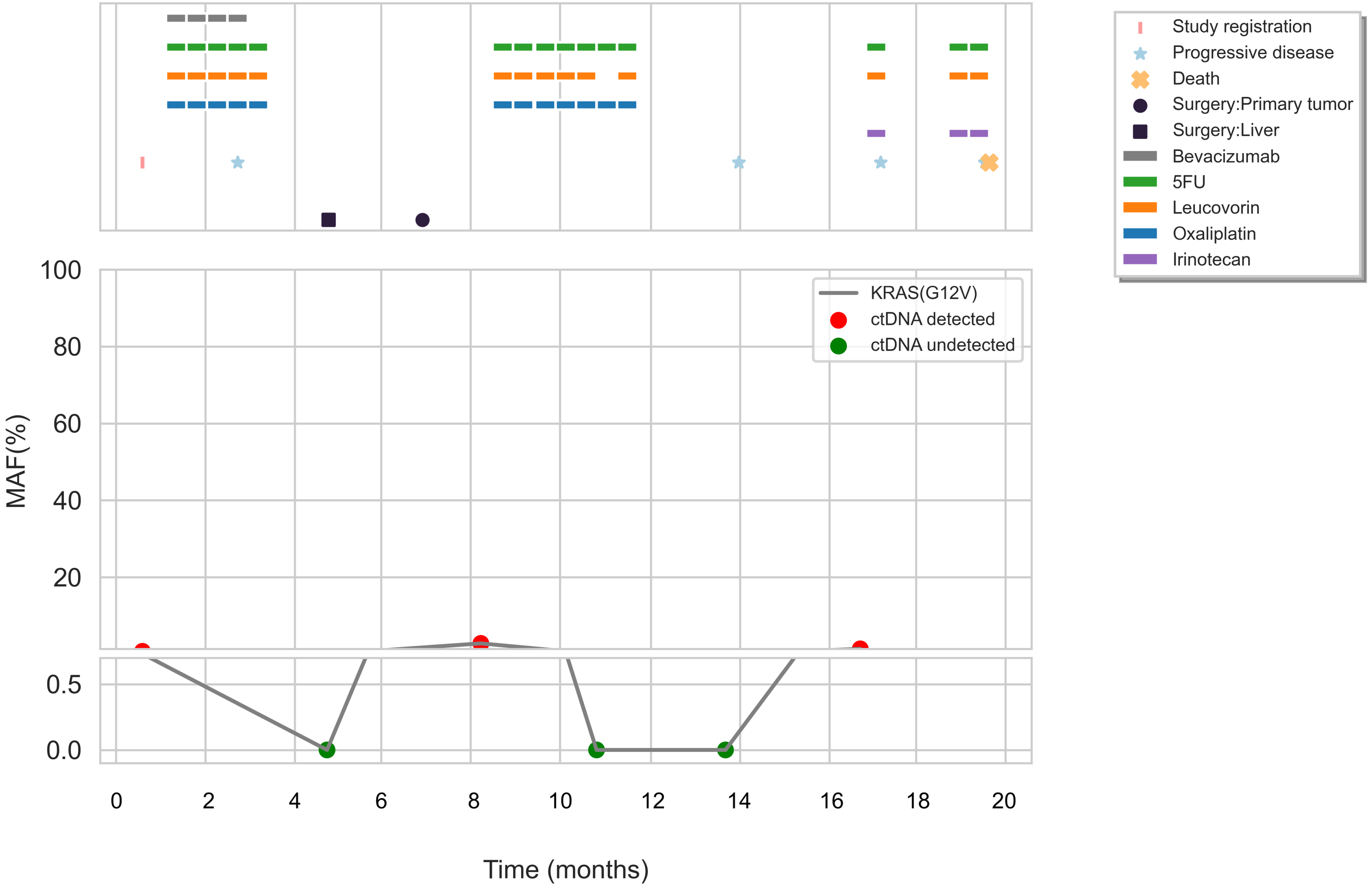

Study registration
Progressive disease
Death
Surgery:Primary tumor
Surgery:Liver
Bevacizumab
5FU
Leucovorin
Oxaliplatin
Irinotecan
KRAS(G12V)
ctDNA detected
ctDNA undetected
MAF(%)
100
80
60
40
20
0.5
0.0
0
2
4
6
8
10
12
14
16
18
20
Time (months)

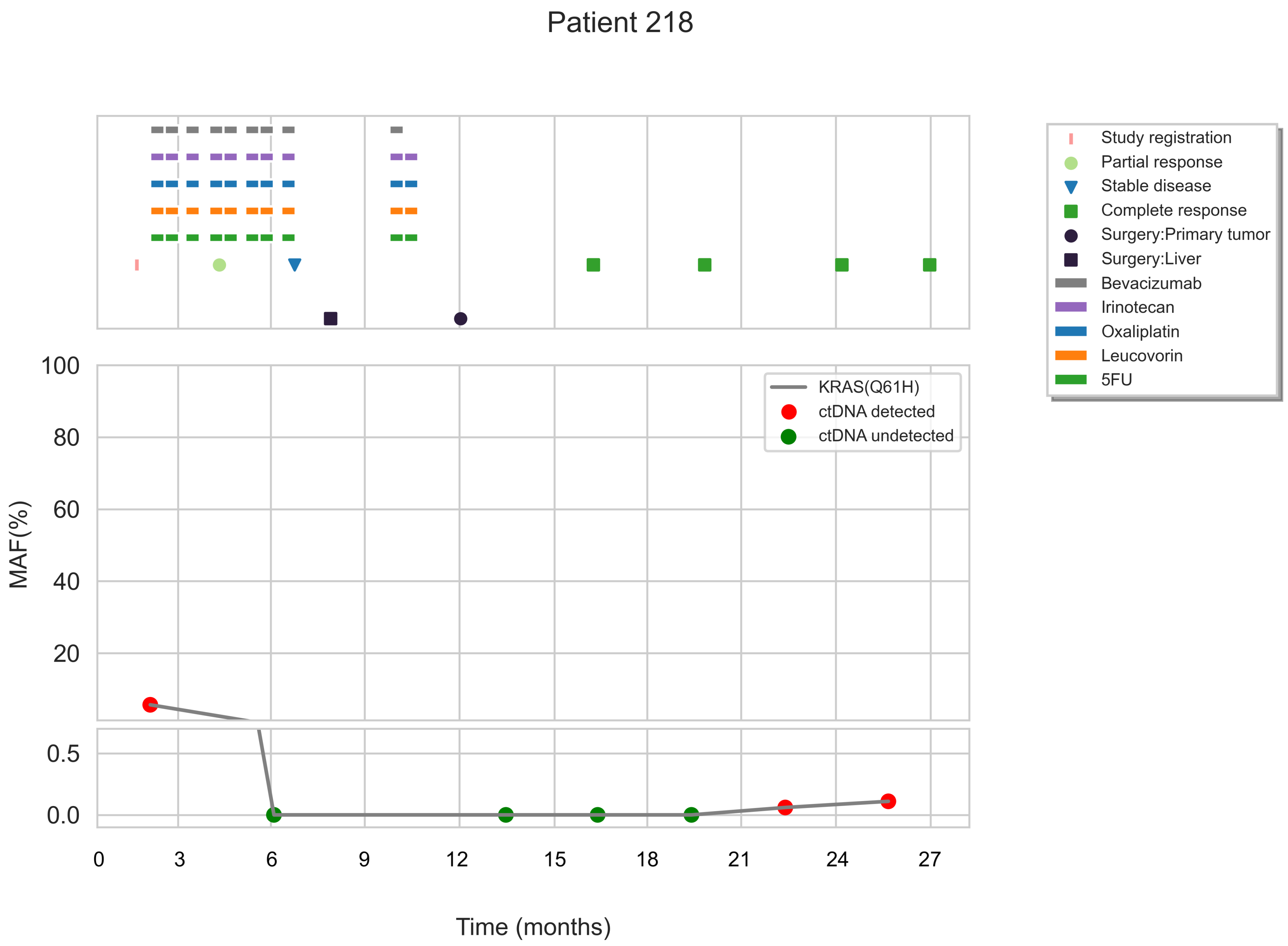
Patient 218
Study registration
Partial response
Stable disease
Complete response
Surgery:Primary tumor
Surgery:Liver
Bevacizumab
Irinotecan
Oxaliplatin
Leucovorin
5FU
KRAS(Q61H)
ctDNA detected
ctDNA undetected
MAF(%)
100
80
60
40
20
0.5
0.0
0
3
6
9
12
15
18
21
24
27
Time (months)

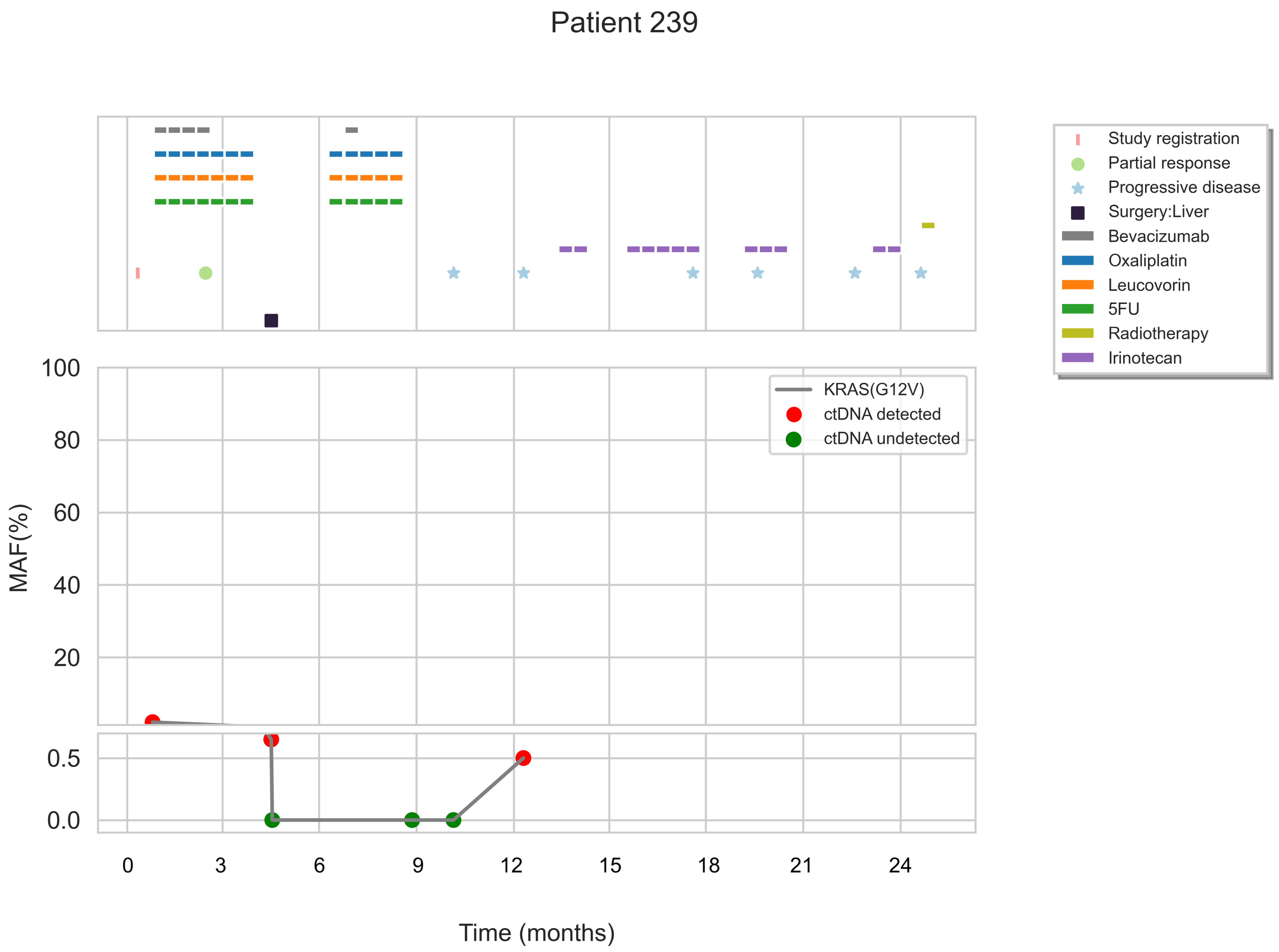

Patient 239
Study registration
Partial response
Progressive disease
Surgery:Liver
Bevacizumab
Oxaliplatin
Leucovorin
5FU
Radiotherapy
Irinotecan
KRAS(G12V)
ctDNA detected
ctDNA undetected
MAF(%)
100
80
60
40
20
0.5
0.0
0
3
6
9
12
15
18
21
24
Time (months)

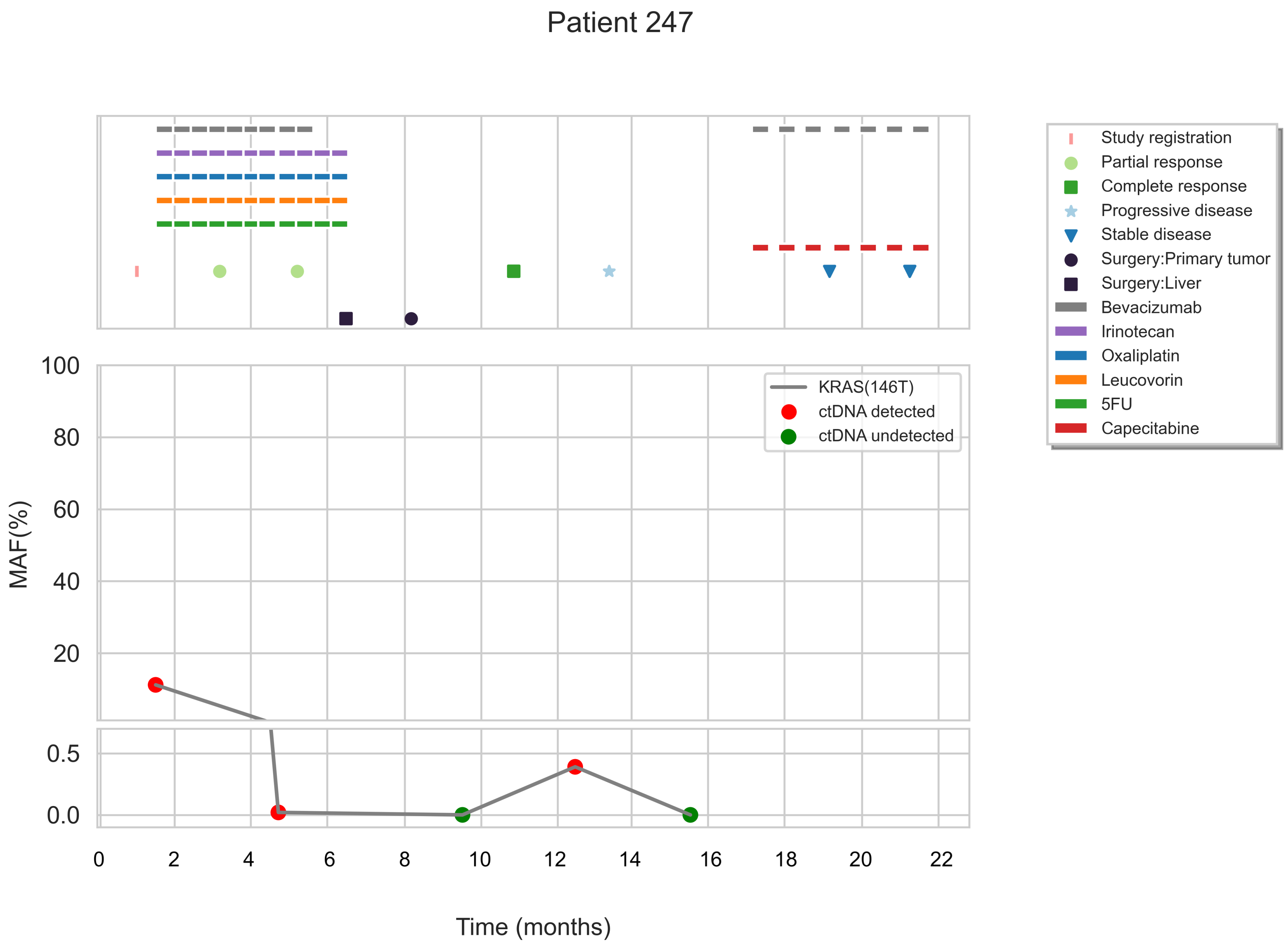

Patient 247
Study registration
Partial response
Complete response
Progressive disease
Stable disease
Surgery:Primary tumor
Surgery:Liver
Bevacizumab
Irinotecan
Oxaliplatin
Leucovorin
5FU
Capecitabine
KRAS(146T)
ctDNA detected
ctDNA undetected
MAF(%)
100
80
60
40
20
0.5
0.0
0
2
4
6
8
10
12
14
16
18
20
22
Time (months)

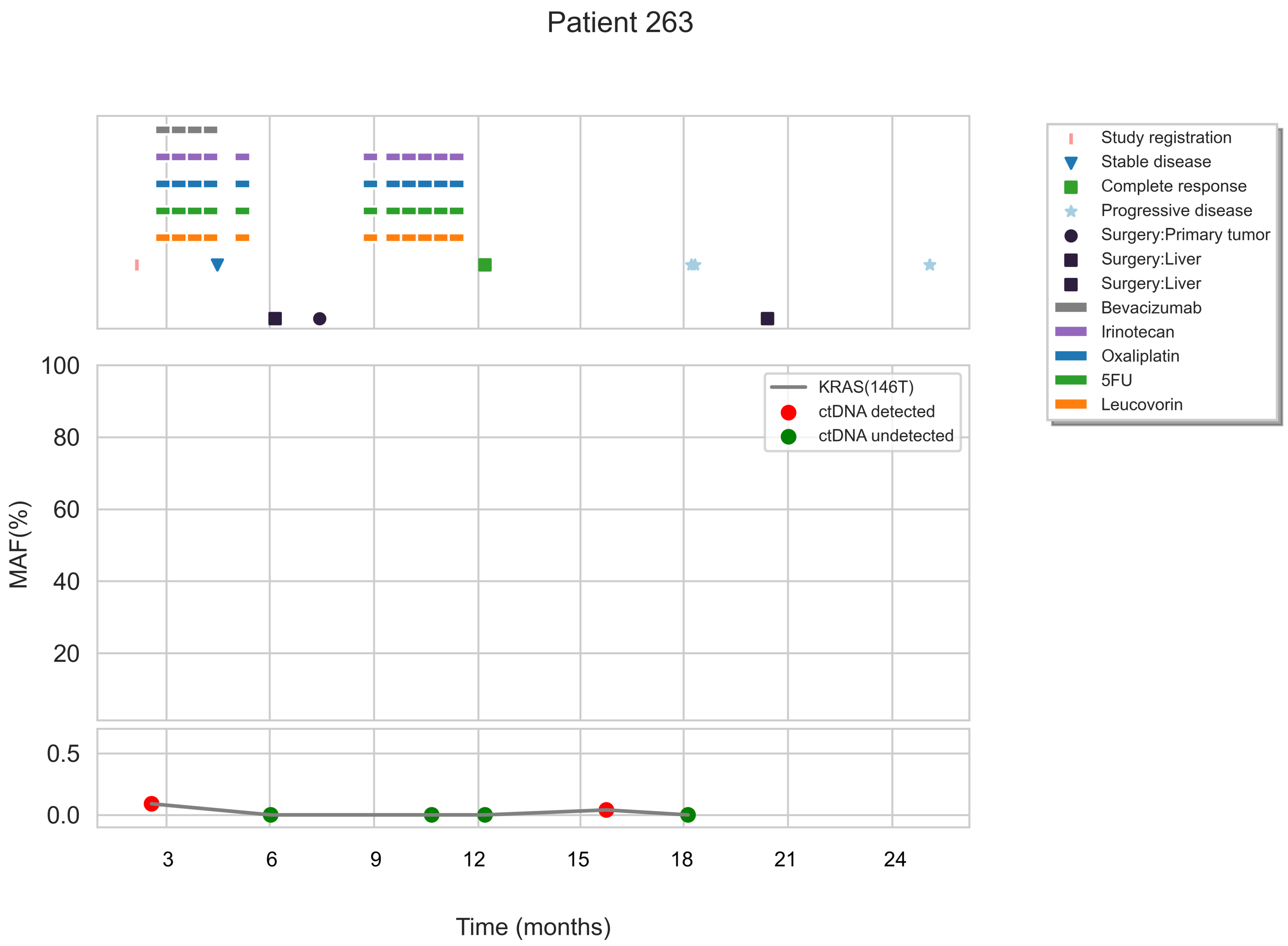
Patient 263
Study registration
Stable disease
Complete response
Progressive disease
Surgery:Primary tumor
Surgery:Liver
Surgery:Liver
Bevacizumab
Irinotecan
Oxaliplatin
5FU
Leucovorin
KRAS(146T)
ctDNA detected
ctDNA undetected
MAF(%)
100
80
60
40
20
0.5
0.0
3
6
9
12
15
18
21
24
Time (months)

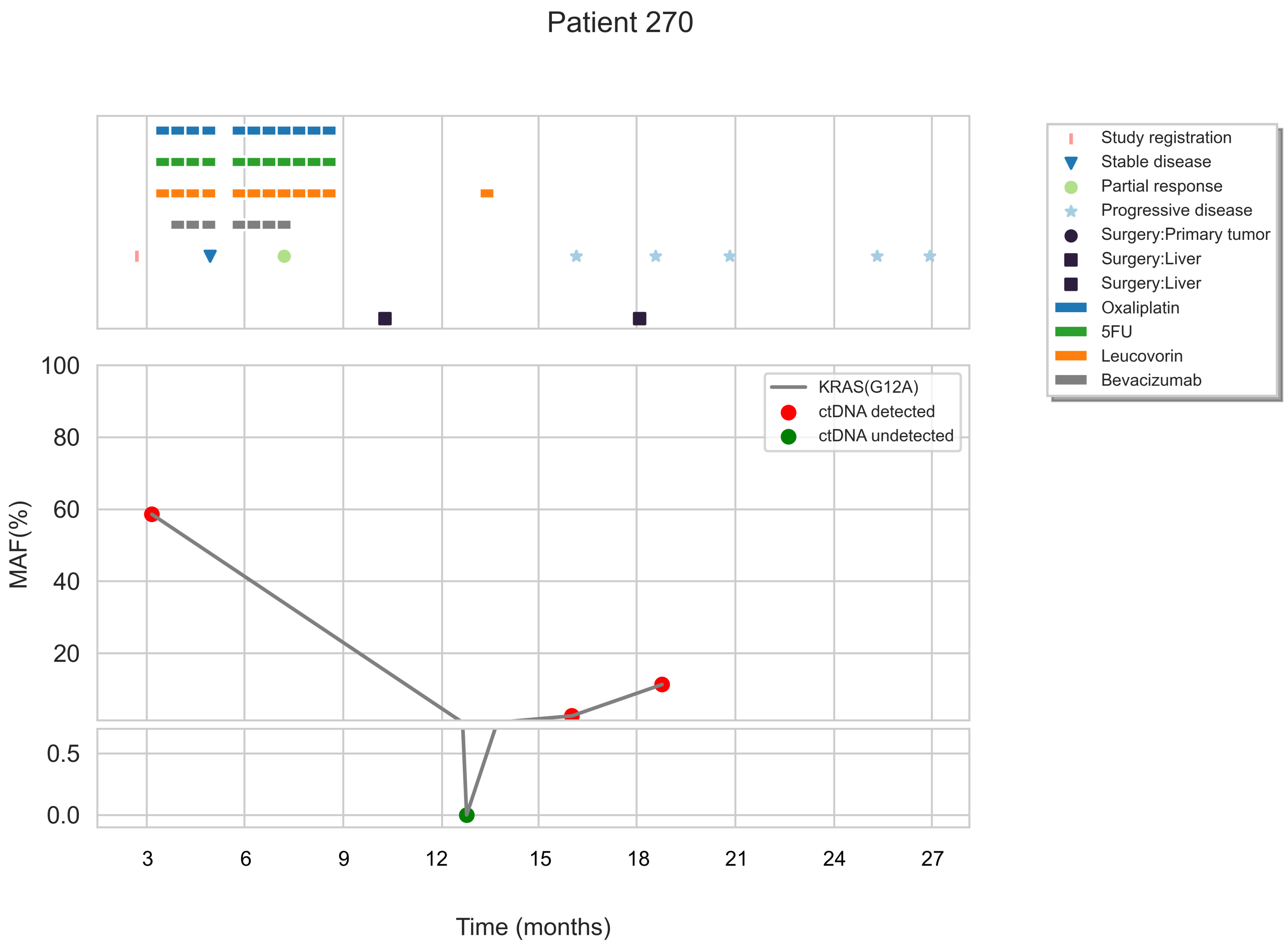
Patient 270
Study registration
Stable disease
Partial response
Progressive disease
Surgery:Primary tumor
Surgery:Liver
Surgery:Liver
Oxaliplatin
5FU
Leucovorin
Bevacizumab
KRAS(G12A)
ctDNA detected
ctDNA undetected
MAF(%)
100
80
60
40
20
0.5
0.0
3
6
9
12
15
18
21
24
27
Time (months)

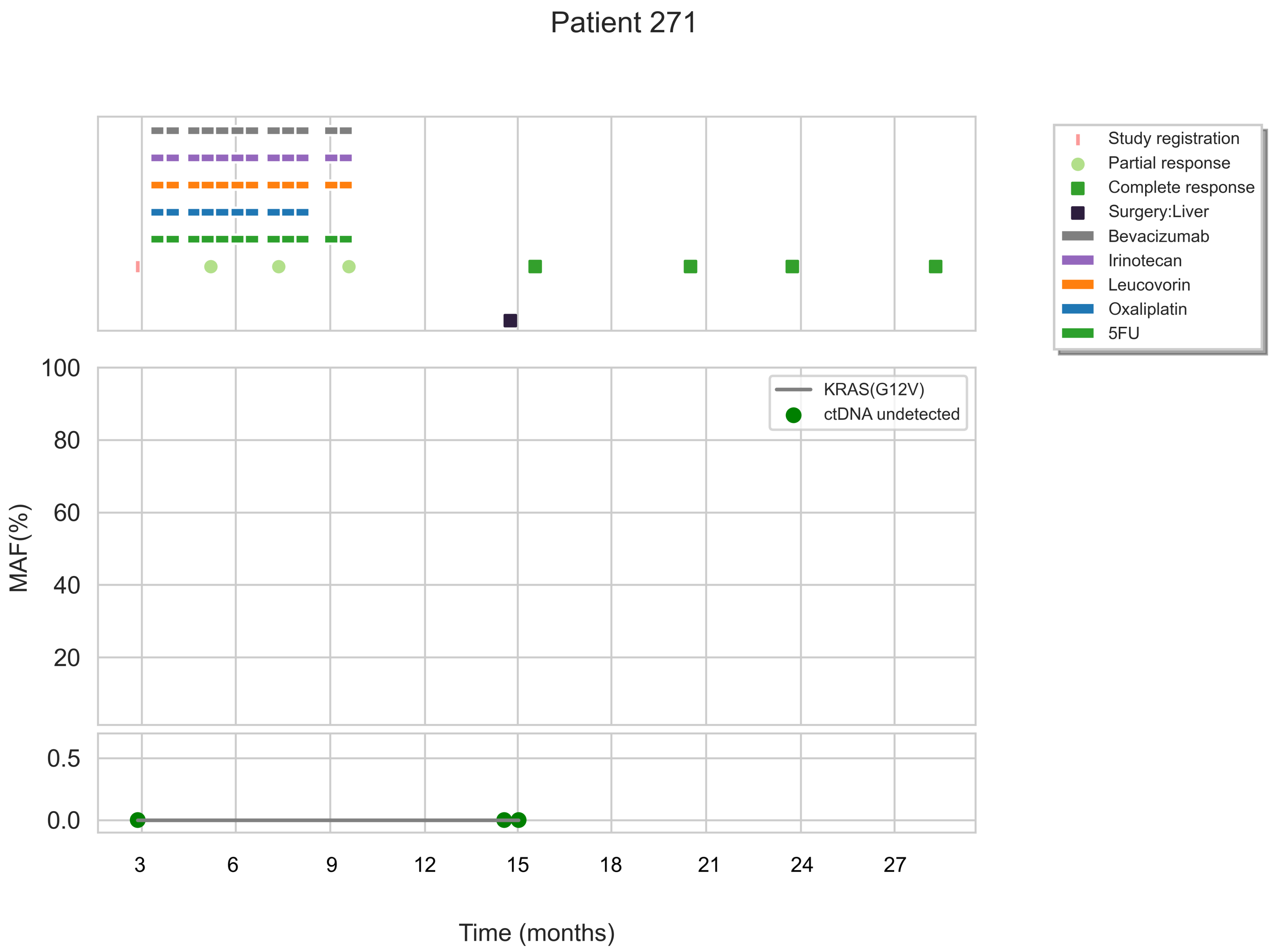

Patient 271
Study registration
Partial response
Complete response
Surgery:Liver
Bevacizumab
Irinotecan
Leucovorin
Oxaliplatin
5FU
KRAS(G12V)
ctDNA undetected
MAF(%)
100
80
60
40
20
0.5
0.0
3
6
9
12
15
18
21
24
27
Time (months)

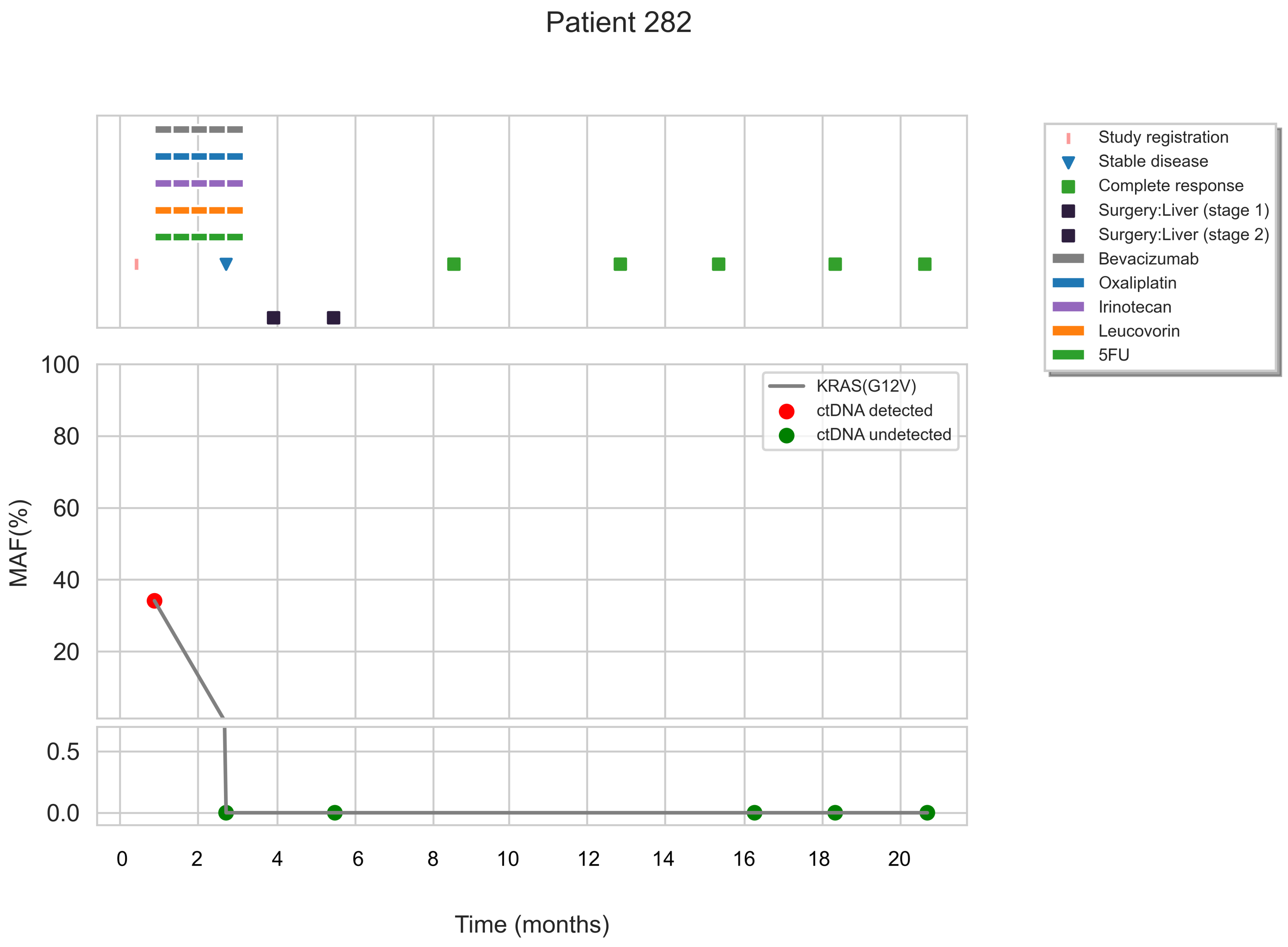

Patient 282
Study registration
Stable disease
Complete response
Surgery:Liver (stage 1)
Surgery:Liver (stage 2)
Bevacizumab
Oxaliplatin
Irinotecan
Leucovorin
5FU
KRAS(G12V)
ctDNA detected
ctDNA undetected
MAF(%)
100
80
60
40
20
0.5
0.0
0
2
4
6
8
10
12
14
16
18
20
Time (months)

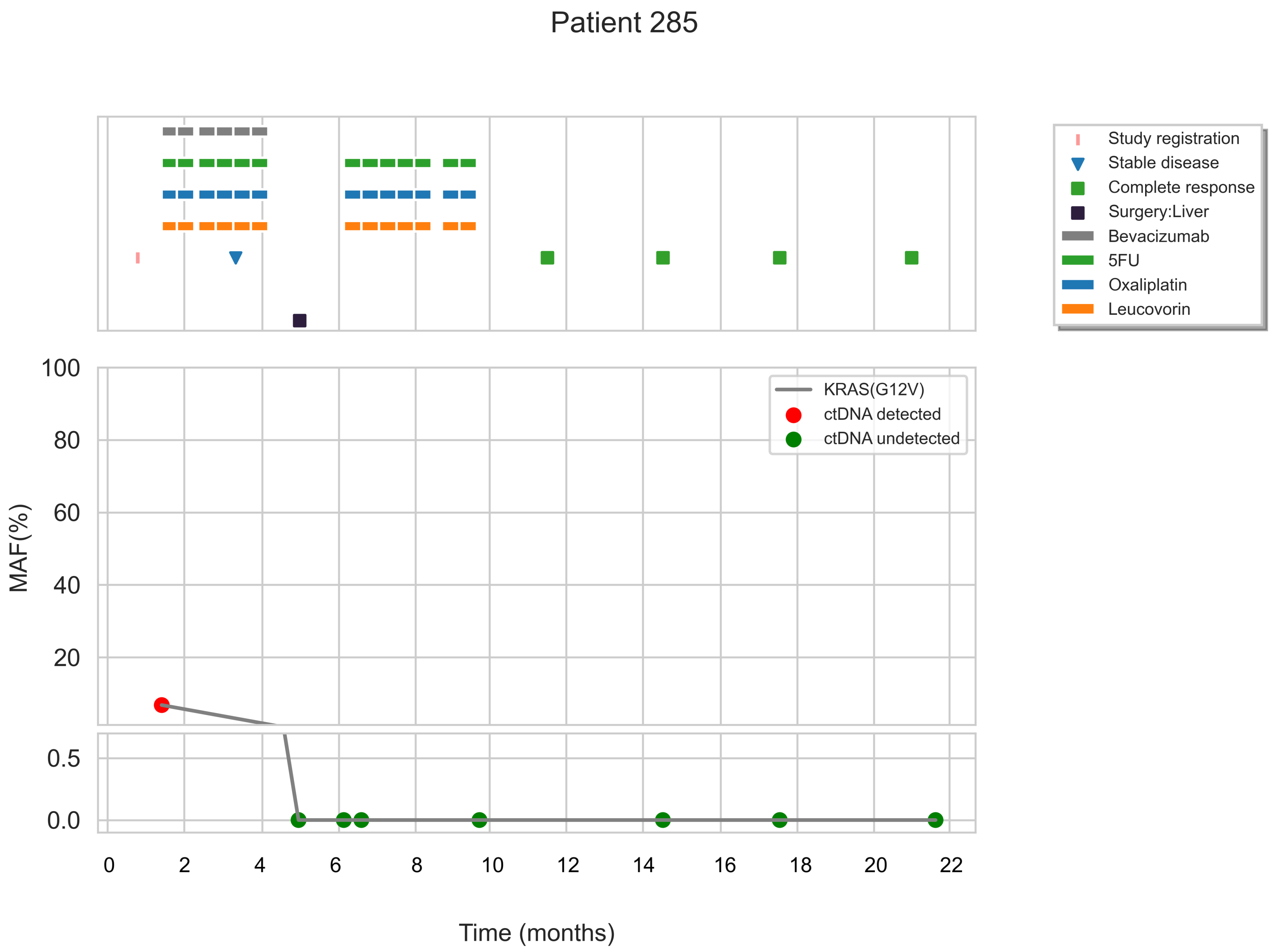

Patient 285
Study registration
Stable disease
Complete response
Surgery:Liver
Bevacizumab
5FU
Oxaliplatin
Leucovorin
KRAS(G12V)
ctDNA detected
ctDNA undetected
MAF(%)
100
80
60
40
20
0.5
0.0
0
2
4
6
8
10
12
14
16
18
20
22
Time (months)

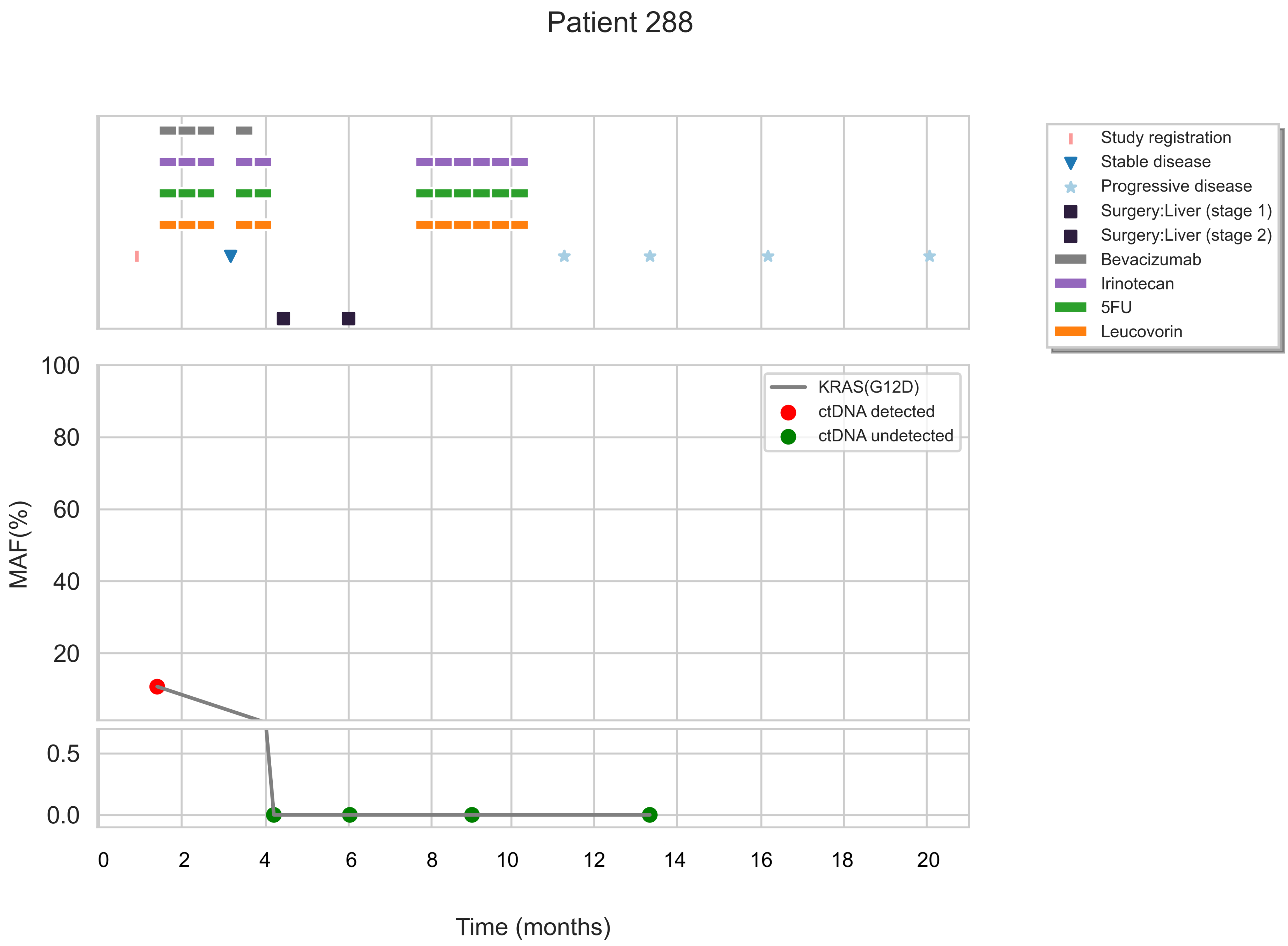

Patient 288
Study registration
Stable disease
Progressive disease
Surgery:Liver (stage 1)
Surgery:Liver (stage 2)
Bevacizumab
Irinotecan
5FU
Leucovorin
KRAS(G12D)
ctDNA detected
ctDNA undetected
MAF(%)
100
80
60
40
20
0.5
0.0
0
2
4
6
8
10
12
14
16
18
20
Time (months)

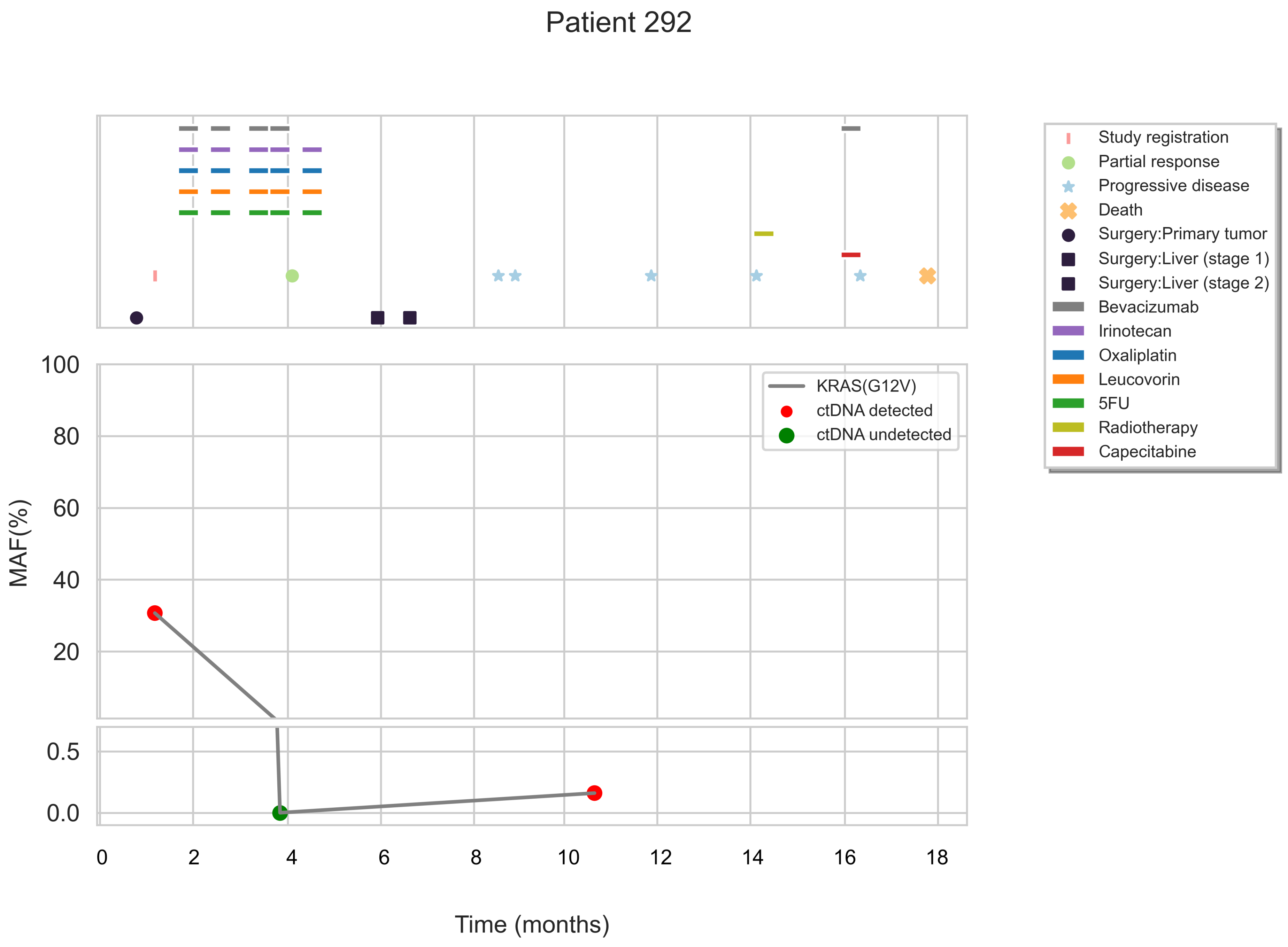

Patient 292
Study registration
Partial response
Progressive disease
Death
Surgery:Primary tumor
Surgery:Liver (stage 1)
Surgery:Liver (stage 2)
Bevacizumab
Irinotecan
Oxaliplatin
Leucovorin
5FU
Radiotherapy
Capecitabine
KRAS(G12V)
ctDNA detected
ctDNA undetected
MAF(%)
100
80
60
40
20
0.5
0.0
0
2
4
6
8
10
12
14
16
18
Time (months)

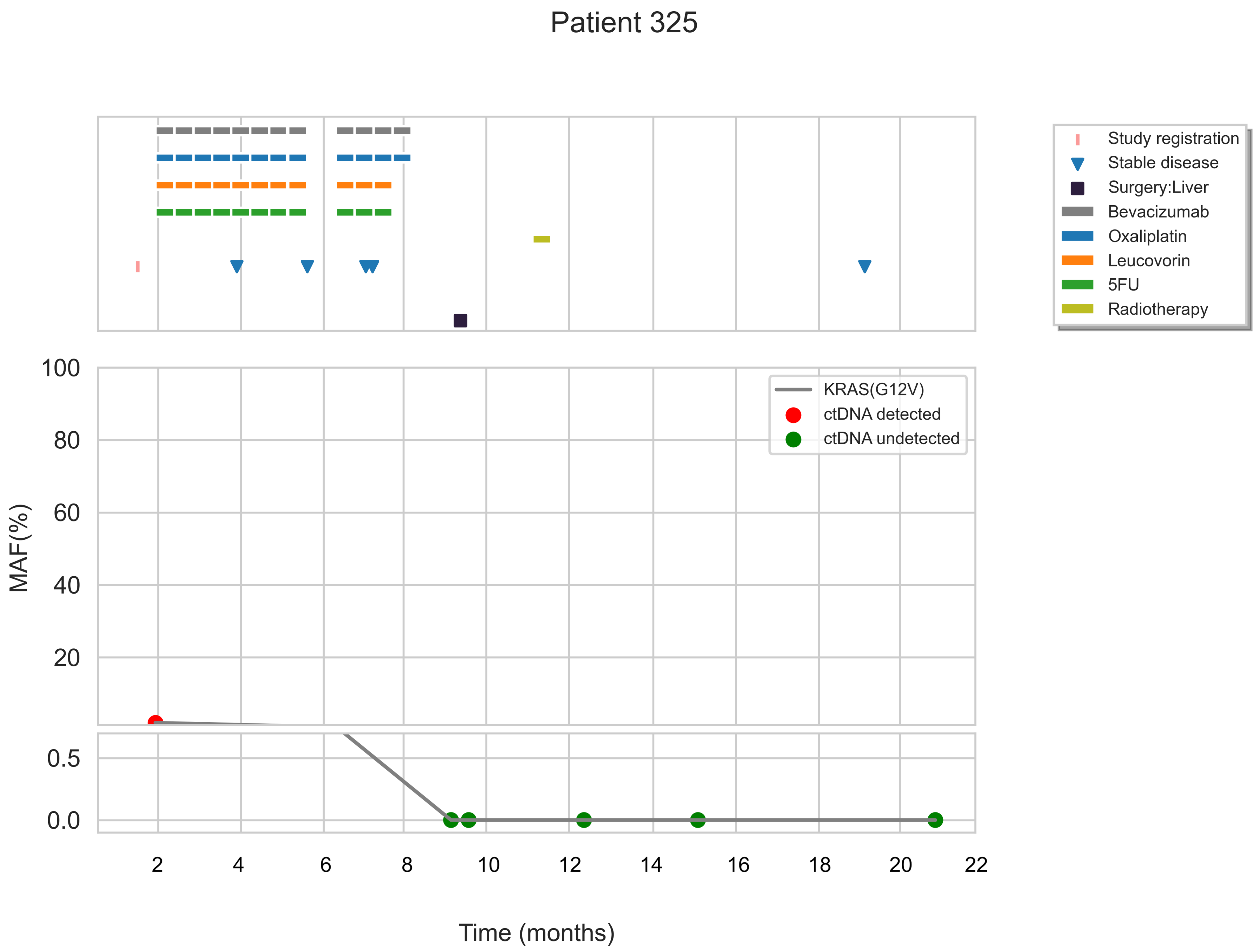

Patient 325
Study registration
Stable disease
Surgery:Liver
Bevacizumab
Oxaliplatin
Leucovorin
5FU
Radiotherapy
KRAS(G12V)
ctDNA detected
ctDNA undetected
MAF(%)
100
80
60
40
20
0.5
0.0
2
4
6
8
10
12
14
16
18
20
22
Time (months)
